# Age‐related macular degeneration and myeloproliferative neoplasms – A common pathway

**DOI:** 10.1111/aos.15247

**Published:** 2022-10-06

**Authors:** Charlotte Liisborg

**Affiliations:** ^1^ Department of Ophthalmology Zealand University Hospital Roskilde Denmark

## Abstract

**Dansk Resumé (Danish summary):**

Aldersrelateret makuladegeneration (AMD) er den hyppigste årsag til uopretteligt synstab og blindhed i højindkomstlande. Det er en progredierende nethindesygdom som gradvist fører til ødelæggelse af de celler som er ansvarlige for vores centralsyn. De tidlige stadier er ofte asymptomatiske, imens senstadie AMD, som opdeles i to former, neovaskulær AMD (nAMD) og geografisk atrofi (GA), begge udviser gradvist synstab, dog generelt med forskellig hastighed. Tidlig AMD er karakteriseret ved tilstedeværelsen af druser og pigmentforandringer i nethinden mens nAMD og GA udviser henholdsvis karnydannelse i og atrofi af nethinden. Ætiologien er multifaktoriel og udover alder omfatter patogenesen miljø‐ og genetiske risikofaktorer. Forskning har specielt fokuseret på lokale forandringer i øjet hvor man har fundet at inflammation spiller en betydelig rolle for udviklingen af sygdommen, men flere studier tyder også på at systemiske forandringer og specielt systemisk inflammation spiller en væsentlig rolle i patogenesen.

De Philadelphia‐negative myeloproliferative neoplasier (MPNs) er en gruppe af hæmatologiske kræftsygdomme med en erhvervet genetisk defekt i den tidlige pluripotente stamcelle som medfører en overproduktion af en eller flere af blodets modne celler. Sygdommene er fundet at udvikle sig i et biologisk kontinuum fra tidligt cancerstadie, essentiel trombocytose (ET) over polycytæmi vera (PV) og endelig til det sene myelofibrose stadie (PMF). Symptomer hos disse patienter skyldes især den ændrede sammensætning af blodet, hyperviskositet, kompromitteret mikrocirkulation og nedsat vævsgennemblødning. Den øgede morbiditet og mortalitet beror i høj grad på tromboembolier, blødninger og leukemisk transformation. En række mutationer som driver MPN sygdommene er identificeret, bl.a. *JAK2V617F*‐mutationen som medfører en deregulering JAK/STAT signalvejen, der bl.a. har betydning for cellers vækst og overlevelse. Et tidligere stort registerstudie har vist at patienter med MPNs har en øget risiko for neovaskulær AMD og et pilotstudie har vist øget forekomst af intermediær AMD. Dette ønsker vi at undersøge nærmere i et større studie i dette Ph.d.‐ projekt. Flere studier har også vist at kronisk inflammation spiller en vigtig rolle for både initiering og udvikling af den maligne celleklon hos MPNs og herfra er en “Human Inflammationsmodel” blevet udviklet. Siden er MPN sygdommene blevet anvendt som “model sygdomme” for en tilsvarende inflammationsmodel for udvikling af Alzheimers sygdom. I dette Ph.d.‐projekt vil vi tilsvarende forsøge at undersøge systemisk inflammation i forhold til forekomst af druser. Det vil vi gøre ved at sammenligne systemiske immunologiske markører som tidligere er undersøgt hos patienter med AMD og sammenligne med MPN. Specielt er vi interesseret i systemiske immunologiske forskelle på patienter med MPN og druser (MPNd) og MPN med normale nethinder (MPNn).

Denne afhandling består af to overordnede studier. I Studie I, undersøgte vi forekomsten af retinale forandringer associeret med AMD hos 200 patienter med MPN (artikel I). Studie II, omhandlede immunologiske ligheder ved AMD og MPN, og var opdelt i yderligere tre delstudier hvor vi undersøgte hhv. systemiske markører for inflammation, aldring og angiogenese (artikel II, III og IV). Vi undersøgte markørerne i fire typer af patienter: nAMD, intermediær AMD (iAMD), MPNd og MPNn. Undersøgelsen af forskelle mellem MPNd og MPNn, vil gøre det muligt at identificere forandringer i immunsystemet som kunne være relevante for AMD‐patogenesen. Vi vil endvidere sammenholde resultaterne for patienter med MPN med patienter som har iAMD og nAMD.

I studie I (Artikel I) fandt vi at patienter med MPN har en signifikant højere prævalens af store druser og AMD tidligere i livet sammenlignet med estimater fra tre store befolkningsundersøgelser. Vi fandt også at forekomst af druser var associeret med højere neutrofil‐lymfocyt ratio, hvilket indikerer et højere niveau af kronisk inflammation i patienterne med druser sammenlignet med dem uden druser.

I studie II (Artikel II, III og IV) fandt vi flere immunologiske forskelle mellem patienter med MPNd og MPNn. Da vi undersøgte markører for inflammation, fandt vi en højere grad af systemisk inflammation i MPNd end MPNn. Dette blev vist ved en højere inflammationsscore (udregnet på baggrund af niveauer af pro‐inflammatoriske markører), en højere neutrofil‐lymfocyt ratio, samt indikationer på et dereguleret komplementsystem. Ved undersøgelse af aldringsmarkører fandt vi tegn på accelereret immunaldring hos MPNd i forhold til MPNn, hvilket kommer til udtryk ved en større procentdel af “effector memory T celler”. Endelig fandt vi en væsentlig lavere ekspression af CXCR3 på T celler og monocytter hos patienter med nAMD sammenlignet med iAMD, MPNd og MPNn. Dette er i overensstemmelse med tidligere studier hvor CXCR3 ekspression er fundet lavere end hos raske kontroller. Derudover fandt vi en faldende CXCR3 ekspression på monocytter over det biologiske MPN‐kontinuum. Disse studier indikerer en involvering af CXCR3 i både nAMD og PMF, begge sygdomsstadier som er karakteriseret ved angiogenese og fibrose.

Ud fra resultaterne af denne afhandling kan vi konkludere at forekomsten af druser og AMD hos MPN er øget i forhold til baggrundsbefolkningen. Endvidere viser vores resultater at systemisk inflammation muligvis spiller en væsentlig større rolle i udviklingen af AMD end tidligere antaget. Vi foreslår derfor en AMD‐model (Figur 18) hvor inflammation kan initiere og accelerere den normale aldersafhængige akkumulation af affaldsstoffer i nethinden, som senere udvikler sig til druser, medførende øget lokal inflammation og med tiden tidlig og intermediær AMD. Dette resulterer i den øgede risiko for udvikling til de invaliderende senstadier af AMD.

**English summary:**

Age‐related macular degeneration (AMD) is the most common cause of irreversible vision loss and blindness in high‐income countries. It is a progressive retinal disease leading to damage of the cells responsible for central vision. The early stages of the disease are often asymptomatic, while late‐stage AMD, which is divided into two entities, neovascular AMD and geographic atrophy (GA), both show vision loss, though generally with different progression rates. Drusen and pigmentary abnormalities in the retina characterise early AMD, while nAMD and GA show angiogenesis in and atrophy of the retina, respectively. The aetiology is multifactorial and, in addition to ageing, which is the most significant risk factor for developing AMD, environmental‐ and genetic risk factors are implicated in the pathogenesis. Research has focused on local changes in the eye where inflammation has been found to play an essential role, but studies also point to systemic alterations and especially systemic inflammation to be involved in the pathogenesis.

The Philadelphia‐negative myeloproliferative neoplasms (MPN) are a group of haematological cancers with an acquired genetic defect of the pluripotent haematopoietic stem cell, characterised by excess haematopoiesis of the myeloid cell lineage. The diseases have been found to evolve in a biological continuum from early cancer state, essential thrombocythemia, over polycythaemia vera (PV), to the advanced myelofibrosis stage (PMF). The symptoms in these patients are often a result of the changes in the blood composition, hyperviscosity, microvascular disturbances, and reduced tissue perfusion. The major causes of morbidity and mortality are thromboembolic‐ and haemorrhagic events, and leukemic transformation. A group of mutations that drive the MPNs has been identified, e.g., the *JAK2V617F* mutation, which results in deregulation of the JAK/STAT signal transduction pathway important, for instance, in cell differentiation and survival. A previous large register study has shown that patients with MPNs have an increased risk of neovascular AMD, and a pilot study has shown an increased prevalence of intermediate AMD. We wish to study this further in a larger scale study. Several studies have also shown that systemic inflammation plays an essential role in both the initiation and progression of the malignant cell clone in MPNs. From this knowledge, a “Human inflammation model” has been developed. Since then, the MPNs has been used as model diseases for a similar inflammation model for the development of Alzheimer's disease. In this PhD project, we would like to investigate systemic inflammation in relation to drusen presence. We will do this by comparing systemic immunological markers previously investigated in patients with AMD and compare with MPN. We are primarily interested in systemic immunological differences between patients with MPN and drusen (MPNd) and MPN with normal retinas (MPNn).

This thesis consists of two main studies. Study I investigated the prevalence of retinal changes associated with AMD and the prevalence of different AMD stages in 200 patients with MPN (paper I). Study II examined immunological similarities between AMD and MPNs. This study was divided into three substudies exploring systemic markers of inflammation, ageing and angiogenesis, respectively. This was done in four types of patients: nAMD, intermediate AMD (iAMD), MPNd and MPNn. Investigating, differences between MPNd and MPNn, will make it possible to identify changes in the immune system, relevant for AMD pathogenesis. Additionally, we will compare patients with MPNs with patients with iAMD and nAMD.

In study I (Paper I), we found that patients with MPNs have a significantly higher prevalence of large drusen and consequently AMD from an earlier age compared to the estimates from three large population‐based studies. We also found that drusen prevalence was associated with a higher neutrophil‐to‐lymphocyte ratio indicating a higher level of chronic low‐grade inflammation in patients with drusen compared to those without drusen.

In study II (papers II, III and IV), we found immunological differences between patients with MPNd and MPNn. When we investigated markers of inflammation, we found a higher level of systemic inflammation in MPNd than MPNn. This was indicated by a higher inflammation score (based on levels of pro‐inflammatory markers), a higher neutrophil‐to‐lymphocyte ratio, and indications of a deregulated complement system. When examining markers of ageing, we found signs of accelerated immune ageing in MPNd compared to MPNn, shown by more senescent effector memory T cells.

Finally, when exploring a marker of angiogenesis, we found a lower CXCR3 expression on monocytes and T cells in nAMD compared to iAMD, MPNd and MPNn, in line with previous studies of nAMD compared to healthy controls. Further, we found decreasing CXCR3 expression over the MPN biological continuum. These studies indicate CXCR3 involvement in both nAMD and PMF, two disease stages characterised by angiogenesis and fibrosis.

From the results of this PhD project, we can conclude that the prevalence of drusen and AMD is increased in patients with MPN compared to the general population. Further, our results show that systemic inflammation may play a far more essential role in AMD pathogenesis than previously anticipated. We, therefore, propose an AMD model (Figure 18) where inflammation can initiate and accelerate the normal age‐dependent accumulation of debris in the retina, which later evolve into drusen, resulting in increased local inflammation, and over time early‐ and intermediate AMD. This results in the increased risk of developing the late debilitating stages of AMD.

## PREFACE

This thesis is the result of my PhD project at the Department of Ophthalmology, Zealand University Hospital, Roskilde and University of Copenhagen, Denmark, conducted from February 2018 to January 2022.

Research has been an educational experience. In the beginning, I felt somewhat confused, but in the process, I felt how I started to have interesting discussions with my supervisors, and I experienced how my academic writing evolved; I became more confident with each published paper. There is no doubt that the journey has been challenging. Occasionally, I have felt overwhelmed by the fields of haematology, ophthalmology, and immunology. From time to time, the amount of information has made me feel lost. Sometimes I would suddenly find myself in a completely different place or subject than intended. I must say that I find the saying “The more I learn, the more I realise how much I do not know” very fitting.

I feel grateful to so many people who have played an important role in helping me write this thesis. First and foremost, I would like to thank my brilliant supervisors, Torben Lykke Sørensen and Hans Carl Hasselbalch, for allowing me to conduct this PhD. I feel fortunate to have had such knowledgeable, enthusiastic professors guiding and advising me; it has been a great pleasure to work with you both!

It is also important for me to thank all the participants in my studies. They have so generously shared personal experience regarding both their disease and their life. I enjoyed the time we spent together, with plenty of time, a luxury not often possible in the everyday clinic, and I hope I succeeded in answering all the great questions they had. I could not have done it without these wonderful people.

Then I would like to thank Ditte Erngaard, Lene Pia Mundt, and all the other employees at the Department of Ophthalmology.

It is also important for me to thank my former PhD colleagues. Yousif Subhi, Marie Krogh Nielsen, Thomas Forshaw, and Christopher Rue Molbech. They have given me great help, especially in the beginning when I was new to research and ophthalmology. Later in my PhD, I have had the pleasure of new PhD colleagues, Anders Jürs, Jenni Martinez Villaruel Hinnerskov and Alexander Kai Thomsen. Thank you for your help, moral support, and great company at the coffee breaks.

In the last year of my PhD, I was offered the opportunity to handle a part‐time job at our research facility. I became part of the clinical trials conducted there, so naturally, I would like to thank my co‐workers, Charlotte Thornbye Larsen, Gitte Henningsen, Birte Bay Højsted, and Sophie Louise Kienle Vinther, for being welcoming and helpful. It was a pleasure to work with you.

I would like to thank the Department of Haematology for their kind help, and I would like to point out a few people. Especially Mette Grymer deserves a big thanks. I am impressed by your handling and overview of all the ongoing research projects and your always positive attitude. I am so grateful for your help contacting patients from your department, making inclusion in my project very smooth. Further, I would like to thank Vibe Skov and Lasse Kjær for their excellent comments on my manuscripts.

I have been fortunate to have many great collaborators during my studies. I would like to thank the Department of Biochemistry for lending me their flow cytometer. Moreover, I would like to thank the Technical University of Denmark, Sanne Schou Berger, and Natasha Morton for helping me with immunoassays, BioXpedia, Charlotte Busch Ahler and Hans Christian Ingerslev for help with SNP analyses.

I am grateful to the assessment committee, Christian Thomas Brandt, Jesper Stentoft and Kai Kaarniranta, for taking the time to evaluate my PhD thesis.

Finally, I would like to thank my amazing family for supporting me along the way.

## INTRODUCTION

### PURPOSE

Senses are essential for human existence. Most people would agree that eyesight is our most precious sense, and its loss leads to devastating consequences. Fundamentally, this PhD project is about contributing to vision loss prevention by obtaining knowledge on a common eye disease known to cause severe irreversible vision loss.

Age‐related macular degeneration (AMD) is a progressive retinal disease leading to damage of the cells responsible for vision. The condition can severely impact an individual's independence and quality of life. The vision loss often causes extensive problems with performing everyday activities such as reading, driving, and recognising faces, and it affects mobility with the risk of isolation. These factors can have an enormous psychological impact, and studies show an increased incidence of depression in patients with AMD (Dawson et al., 2014; Taylor et al., 2016).

With the rising life expectancy, the prevalence of AMD is expected to increase, impacting the individuals afflicted and health care systems worldwide, adding to an already existing burden.

Despite extensive research in AMD, the exact pathophysiology remains unknown. In recent years, a great deal of focus has been on the immune system and inflammation's role in the pathogenesis of the disease.

Although the existing therapy for the wet form of late‐stage AMD, intravitreal anti‐vascular endothelial growth factor injections, has improved the visual outcomes for many patients, the need for a better understanding and treatment of the disease is evident. We do not have a treatment for the dry form of AMD, and we only treat the disease when an individual has reached the late stage of the disease, where vision loss is already present.

In this PhD project, the aim was to acquire knowledge on AMD by studying a completely different patient group showing alterations in the immune system, signs of massive inflammation, and most importantly, an increased AMD risk. The diseases of interest are the chronic myeloproliferative neoplasms (MPNs). The approach was to identify changes in the immune system common to AMD and MPNs and investigate if MPN patients with signs of AMD are immunologically different from MPN patients with normal retinas. The results may reveal more about the pathogenesis of AMD.

### BACKGROUND

This section will present the background information relevant to this PhD project's studies, first, with a description of the retina, AMD, and MPNs. Hereafter, “the connection” between MPNs and AMD will be described, and three relevant features: inflammation, ageing and angiogenesis.

#### The retina

**FIGURE 1:**
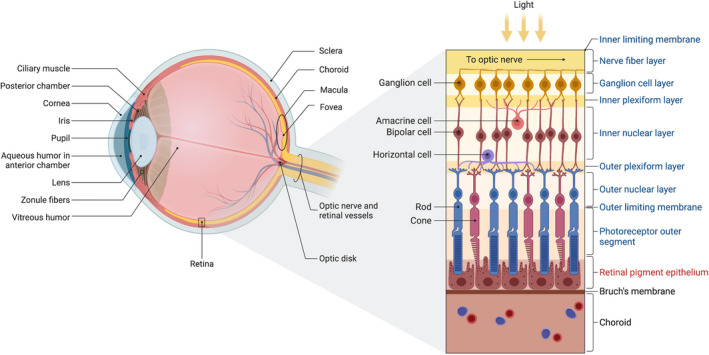
Anatomy of the human eye and the layers of the retina. Left: Eyeball anatomy – sagittal view. Right: Enlargement and anatomy of the retina, showing the ten layers – the neuroretina in blue text and the RPE in red. *Created with BioRender*.

The retina is the innermost, light‐sensitive layer covering the posterior two‐thirds of the eye (Figure 1). We separate it into ten layers; the nine innermost layers comprise the neuroretina, and the outer layer is the retinal pigment epithelium (RPE). The neuroretina contains the two types of light‐sensitive photoreceptors, rods, and cones, which detect light under different conditions. The cones function in bright light and are responsible for high acuity and colour vision. The rods are more sensitive and work in low light settings, thereby responsible for night‐vision. The light stimuli of the photoreceptors are transformed into nerve signals and transmitted via other neurons of the neuroretina, the bipolar cells and ganglion cells. The ganglion cells' nerve fibres form the optic nerve that transmits the transformed light stimuli to the brain to be processed into visual images. The neuroretina also contains modulating nerve cells (horizontal and amacrine cells) and glial cells responsible for supportive and immune functions (microglia and Müller cells) (Kolb, 2007).

The RPE is a monolayer of pigmented cells nourishing and supporting photoreceptors. Tight‐junctions between RPE‐cells form the outer blood‐retina‐barrier (BRB). The Bruch's membrane (BM) sits between the RPE and the underlying choroid acting as a supportive barrier. The BM restricts cellular migration and facilitates the flow of nutrients, oxygen, and waste products between the choroid and the RPE. The BM consist of five layers: the innermost layer composes the basement membrane of the RPE, the outermost layer the basement membrane of the choriocapillaris, which is the part of the choroid where fenestrated capillaries are located. Tight‐junctions between these epithelial capillary cells of the choriocapillaris form the inner BRB. The BRB functions to limit and regulate the movement of molecules and immune cells to the subretinal space (between the RPE and the photoreceptors). Thereby, the BRB have a vital function in supporting and maintaining retinal immune privilege. The choroid layers are from the retinal site inwards out; the outer layer of BM, the choriocapillaris described above, Sattler's layer and Haller's layer, containing medium diameter vessels and larger diameter vessels, respectively (Kolb, 2007).

The macula (Figure 1) is a circular region of the retina approximately 5.5mm in diameter, providing central vision. It is located at the posterior pole of the eye temporally to the optic disc (The optic disc is the point where nerve fibres of the ganglion cells exit the eye to form the optic nerve). In the very centre of the macular region is the fovea, with a diameter of 1.5mm. Only cone photoreceptors are present in the fovea, and it is the part of the retina where visual acuity is highest. The retina outside the macula provides peripheral vision and is used when moving or orientating (Kolb, 2007).

It is possible to look directly at the retina through the pupil with direct ophthalmoscopy and different imaging techniques. The methods section will briefly describe the imaging techniques used in this study.

#### Age‐related macular degeneration

##### Presentation and classification

**FIGURE 2:**
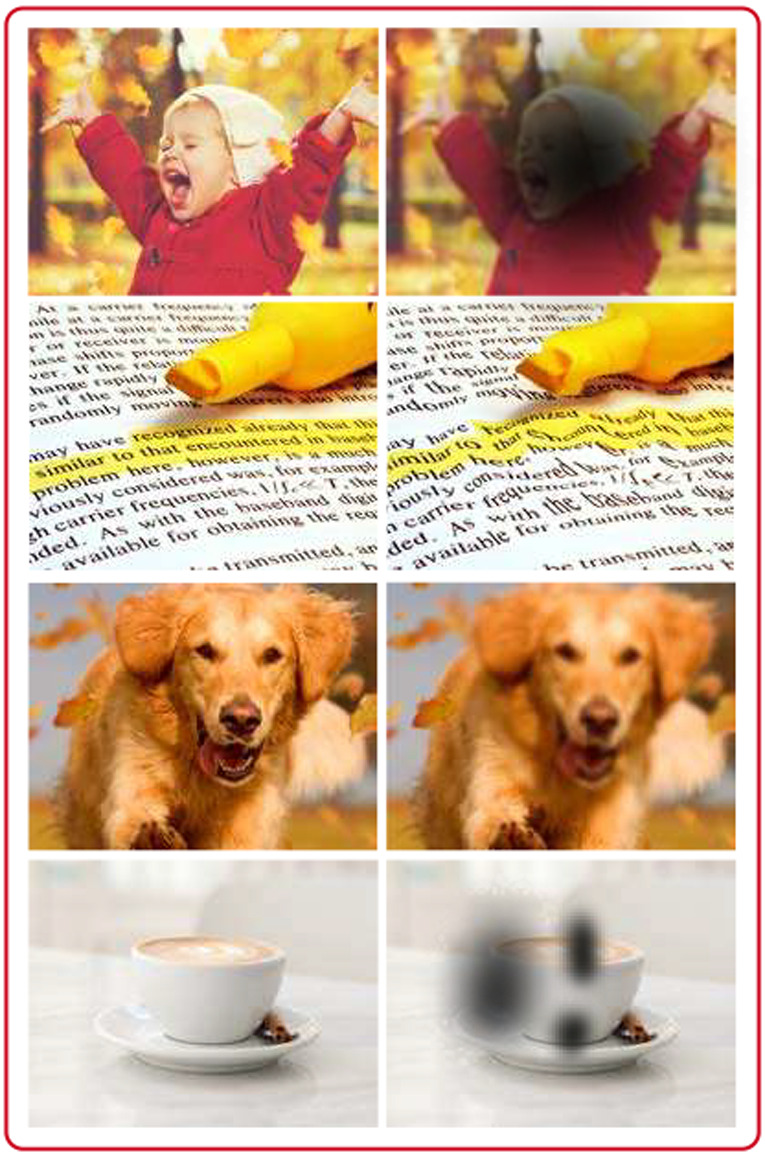
visual distortions seen in patients with age‐related macular degeneration. *Created with Adobe Illustrator*. Royalty free photos from dreamstime.com.

Age‐related macular degeneration (AMD) is a degenerative disease of the retina, especially the macular area. The disease affects the photoreceptors, the RPE, BM and the choroid and results in progressive loss of central vision. Common symptoms are visual distortions such as metamorphopsia (straight lines appear wavy), blurred vision and scotomas (blind spots) (Figure 2). These symptoms are seen primarily in the later stages of AMD.

The hallmark sign of AMD is drusen which are present at all AMD stages. These are white‐yellowish deposits of extracellular material (lipids and proteins) accumulating between the RPE and BM. A specific form of drusen called subretinal drusenoid deposits (or reticular pseudodrusen) is seen between the RPE and the photoreceptors and is associated with a greater risk of AMD (Spaide et al., 2018). Based on drusen presence, size and number and some other clinical characteristics described below, we classify AMD in different stages: early‐, intermediate‐ and late AMD (Ferris et al., 2013) (Table 1). Late‐stage AMD can be further divided into two morphological sub‐types: the neovascular form (also called wet AMD) and the atrophic form called geographic atrophy (GA).

**TABLE 1:** Classification system introduced by the Beckman Initiative for Macular Research Classification Committee (Ferris et al., 2013)

**AMD Classification (Beckmann)**	
Classification stage	Definition (fundus lesion within two disc diameters of the fovea)
No disease	No drusen, no pigment changes
Normal ageing	None or few drupelets (small drusen <63 μm) No AMD pigmentary abnormalities
Early AMD	Medium‐sized drusen >63 and ≤125 μm
No AMD pigmentary abnormalities	
Intermediate AMD	Large drusen > 125 μm and/or AMD pigmentary abnormalities
Late AMD ‐ 2 forms	Neovascular AMD (neovascularisations) Geographic atrophy (retinal atrophy)

In early AMD (eAMD), only medium‐sized drusen are observed. The criteria for intermediate AMD are met when both drusen and pigmentary abnormalities are seen (either hypo‐ or hyperpigmented areas of the retina) or if large drusen are present. The late neovascular form (nAMD) is defined by abnormal blood vessel growth, either above the RPE (classic) or below (occult). The vessels stem from either the choriocapillaris (choroidal neovascularisations – CNV) or the retinal vasculature (retinal angiomatous proliferations – RAP). The atrophic form, GA, is characterised by areas of retinal atrophy, RPE and photoreceptor loss or dysfunction. The early stages of AMD often progress slowly and asymptomatically, while severe and rapid vision loss are seen in nAMD due to leaking of the new fragile blood vessels causing irreversible damage to the photoreceptors. In GA, the areas of atrophy typically appear in the perifoveal area. Moreover, the atrophies often progress slowly with later involvement of the fovea and devastating vision loss (Fleckenstein et al., 2021).

No treatment is available for eAMD, iAMD and GA (together referred to as dry AMD), but nAMD are treated with anti‐vascular endothelial growth factor (anti‐VEGF) therapy, given as intravitreal injections. This therapy is not curative but is often effective in preventing severe vision loss (Fleckenstein et al., 2021).

The natural history and prognosis of nAMD, if left untreated, is severe visual loss with an average visual acuity loss of around four lines (measured by logarithm of the minimum angle of resolution – logMAR) within two years of disease onset (Wong et al., 2008). In GA, the progression rate is very variable between individuals (Fleckenstein et al., 2018).

##### Epidemiology

A systematic literature review in 2013 estimated the worldwide prevalence of AMD to be 8.7% in the age range 45 to 85 years (including all stages from early to late AMD) (Wong et al., 2014). An estimation shows that 196 million people around the globe are affected by AMD, and due to the rapidly growing and ageing population, the number is expected to increase to 288 million by 2040. Worldwide, AMD accounts for 5.6% of blindness cases and 3% of moderate to severe vision impairment (MSVI), preceded by other eye conditions; cataract, glaucoma and uncorrected refractive errors as the leading causes (GBD 2019 Blindness and Vision Impairment Collaborators & Vision Loss Expert Group of the Global Burden of Disease Study, 2021). In developed countries, AMD is the second most common cause of blindness in the elderly after cataract a‑nd the third most common reason for MSVI following uncorrected refractive errors and cataract (Bourne et al., 2018). The prevalence of AMD in Denmark is shown in Table 2.

**TABLE 2:** Prevalence of AMD in Denmark

**AMD in Denmark** (Sedeh et al., 2017) In Denmark, with a population of 5.8 million people, estimation shows that around 33,000 people have neovascular AMD and 23,000 have geographic atrophy in 2020. The majority are people aged 85 years or older. These numbers are expected to increase to approximately 58,000 for neovascular AMD and 41,000 for geographic atrophy by 2040.

Systematic meta‐analyses (Chakravarthy et al., 2010; Heesterbeek et al., 2020) summarise the risk factors for late AMD. Morphological risk factors are the presence of drusen, drusen size and type, and pigmentary abnormalities. Further, vascular changes in the choroidal vasculature are a risk factor. The most important demographic risk factor is age. Population studies show a prevalence of early AMD of 3.5% in the age group 55‐59 years and increasing to about 17.6% in people 85 years and older. The corresponding numbers for late AMD in the same age groups are 0.1% and 9.8%. Other strong demographic risk factors are tobacco use, previous cataract surgery, and a family history of AMD and moderate risk factors are hypertension, a history of cardiovascular diseases, higher BMI and higher plasma fibrinogen (Chakravarthy et al., 2010). Several other risk factors have been studied, but the evidence in the literature remains variable. For example, studying dietary factors and physical exercise is more complicated, but associations with AMD have also been found here. Genetic factors play an important role in the development of AMD, especially variants in genetic loci encoding complement system components show a strong association.

In summary, AMD is a complex and multifactorial disease, and the pathogenesis is not known in detail. The disease is caused by an interaction of age, genetic susceptibility, environmental‐ and demographic factors. In this thesis, the focus will be on the impact of ageing, inflammation, and angiogenesis (measured in the systemic circulation) described further in sections “inflammation”, “ageing”, and “angiogenesis” on page 13–17.

##### Treatment

As mentioned above, we treat nAMD with intravitreal anti‐VEGF injections, and since the introduction of this treatment, the prognosis for many AMD patients has markedly improved. The treatment is not curative, and the patients need continuous injections with many hospital visits and variable treatment responses.

Some studies show the benefit of vitamin supplements for patients with intermediate AMD or late AMD in only one eye. Vitamin supplements with the combination of vitamin C, vitamin E, lutein, zeaxanthin, zinc and copper may help slow disease progression (Chew et al., 2013).

#### Myeloproliferative neoplasms

##### Presentation and Classification

**FIGURE 3:**
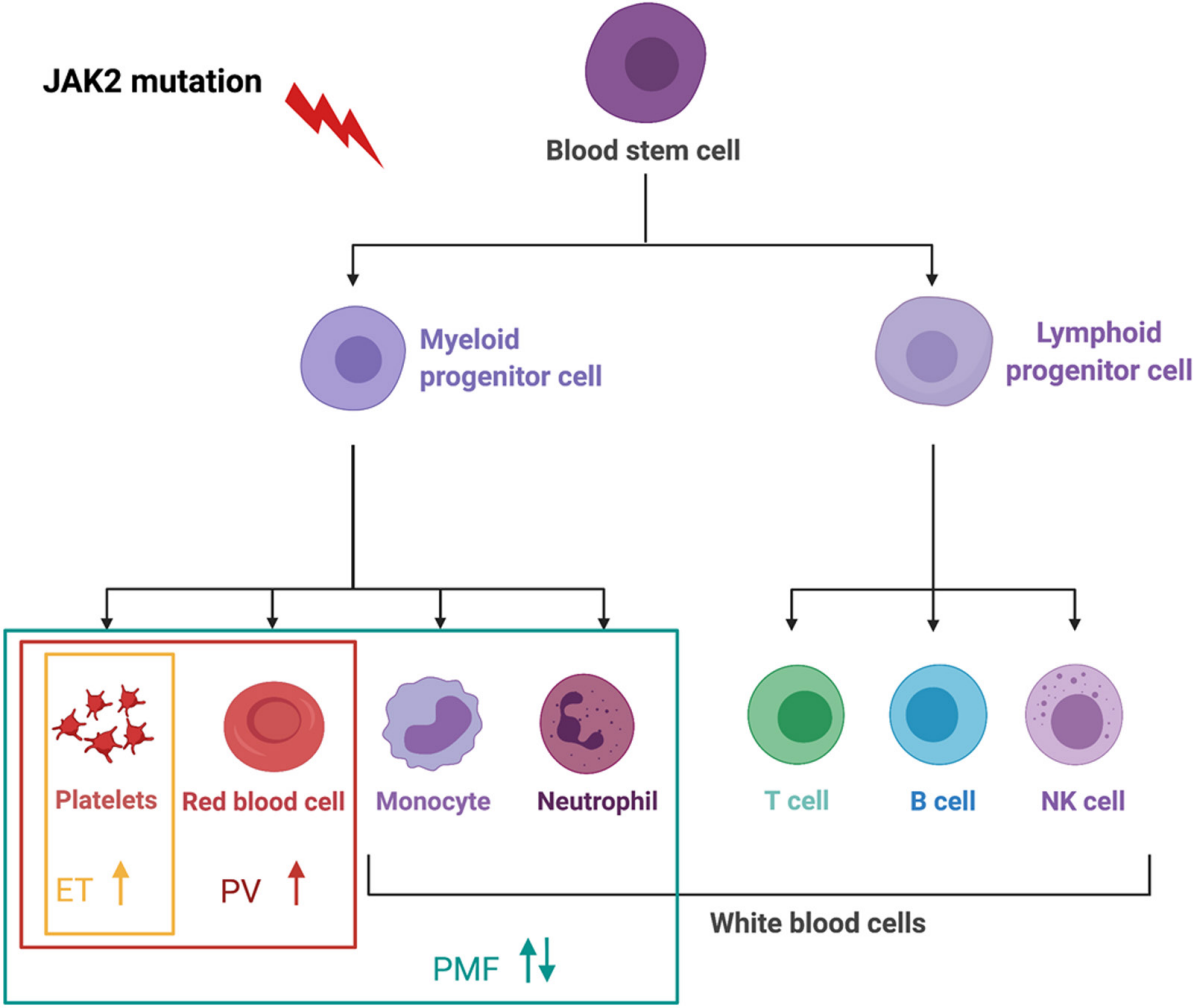
Simplified diagram of haematopoiesis and simplified characteristics view of the MPNs. The *JAK2V617* mutation (or other relevant mutations) result in an abnormal haematopoietic stem cell, leading to excess haematopoiesis of the myeloid lineage. In ET, thrombocytosis is the dominating sign, but leukocytosis is also seen. The classical characteristic of PV is erythrocytosis, but nearly all patients develop leukocytosis and thrombocytosis if not already present at diagnosis. The classic PMF patient characteristics are anaemia, variable leukocyte and platelet count changes, bone marrow fibrosis and splenomegaly. *Created with BioRender*.

The myeloproliferative neoplasms (MPNs) are a group of haematological cancers (Table 3). They arise from the clonal proliferation of an abnormal pluripotent haematopoietic stem cell and are characterised by excess haematopoiesis of the myeloid cell lineage (Figure 3). Three major subtypes of MPNs are the Philadelphia‐chromosome negative chronic myeloproliferative neoplasms, often referred to as the “classic MPNs” (highlighted in Table 3). The classic MPNs are this project's focus and will, throughout the thesis, be referred to as the MPNs.

**TABLE 3:** 2016 World Health Organisation (WHO) Classification of Tumours of Haematopoietic and Lymphoid Tissues, with the Philadelphia‐negative myeloproliferative neoplasms in bold

**Myeloproliferative neoplasms**
Chronic myeloid leukaemia ‐ CML (BCR‐ABL1‐positive)
Chronic neutrophilic leukaemia
**Polycythemia vera – PV**
**Essential thrombocythemia – ET**
Chronic eosinophilic leukaemia, NOS
Myeloproliferative neoplasms, Unclassifiable
**Primary myelofibrosis, pre‐fibrotic/early‐stage ‐ pre‐PMF**
**Primary myelofibrosis, overt fibrotic stage ‐ PMF**

The MPNs are closely related and are considered a biological continuum with an overlapping clinical presentation from the early cancer stage; essential thrombocythemia (ET) over polycythaemia vera (PV), to the late advanced primary myelofibrosis stage (PMF) (Barosi et al., 2012; Larsen et al., 2007; Peniket, 2002). Primary myelofibrosis can be further divided into pre‐fibrotic/early‐stage PMF and overt fibrotic PMF (Spivak, 2017). The diagnosis of the subtypes is based upon bone marrow morphology, a complete blood count, and specific driver mutations. For diagnosis of PMF, the presence of palpable splenomegaly and high serum lactate dehydrogenase levels (LDH) are also evaluated (Barbui et al., 2018).

The discovery of the Janus kinase 2 *(JAK2) V617F* mutation and later other driver mutations (*JAK2* exon 12, *MPL* and *CALR*) has markedly enhanced the understanding of the biology of these disorders. The *JAK2* mutation is present in 95‐98% of patients with PV, 50‐60% in ET and 55‐65% in PMF. *CALR* and *MPL* are not seen in PV, but the *CALR* mutation is present in 20‐25% of patients with ET and PMF and the MPL mutation in 3‐4% of patients with ET and 6‐7% in PMF (Tefferi & Pardanani, 2015). The mutations all share the characteristic of affecting the JAK‐STAT signal transduction pathway generating the classic MPN phenotype, with activation and proliferation of the haematopoietic progenitor cell, and uncontrolled production of different terminal blood cell quantities, depending on MPN subtype and mutation type (Shallis et al., 2020). In the *JAK2* and *CALR* positive, a steady increase in the mutational allele burden (“tumour” burden) is seen over the biological continuum for MPNs from the early‐stage disease to the advanced burned out PMF stage (Cavalloni et al., 2017; Hasselbalch, 2013).

In patients with ET, thrombocytosis predominates, but a subset of patients shows leukocytosis too. In patients with PV, the hallmark is erythrocytosis, but if not already present at diagnosis, nearly all patients develop leukocytosis and thrombocytosis as well. The classic PMF characteristics are anaemia, variable leukocyte and platelet count changes, bone marrow fibrosis, and splenomegaly (Spivak, 2017; Tefferi & Pardanani, 2015).

**FIGURE 4:**
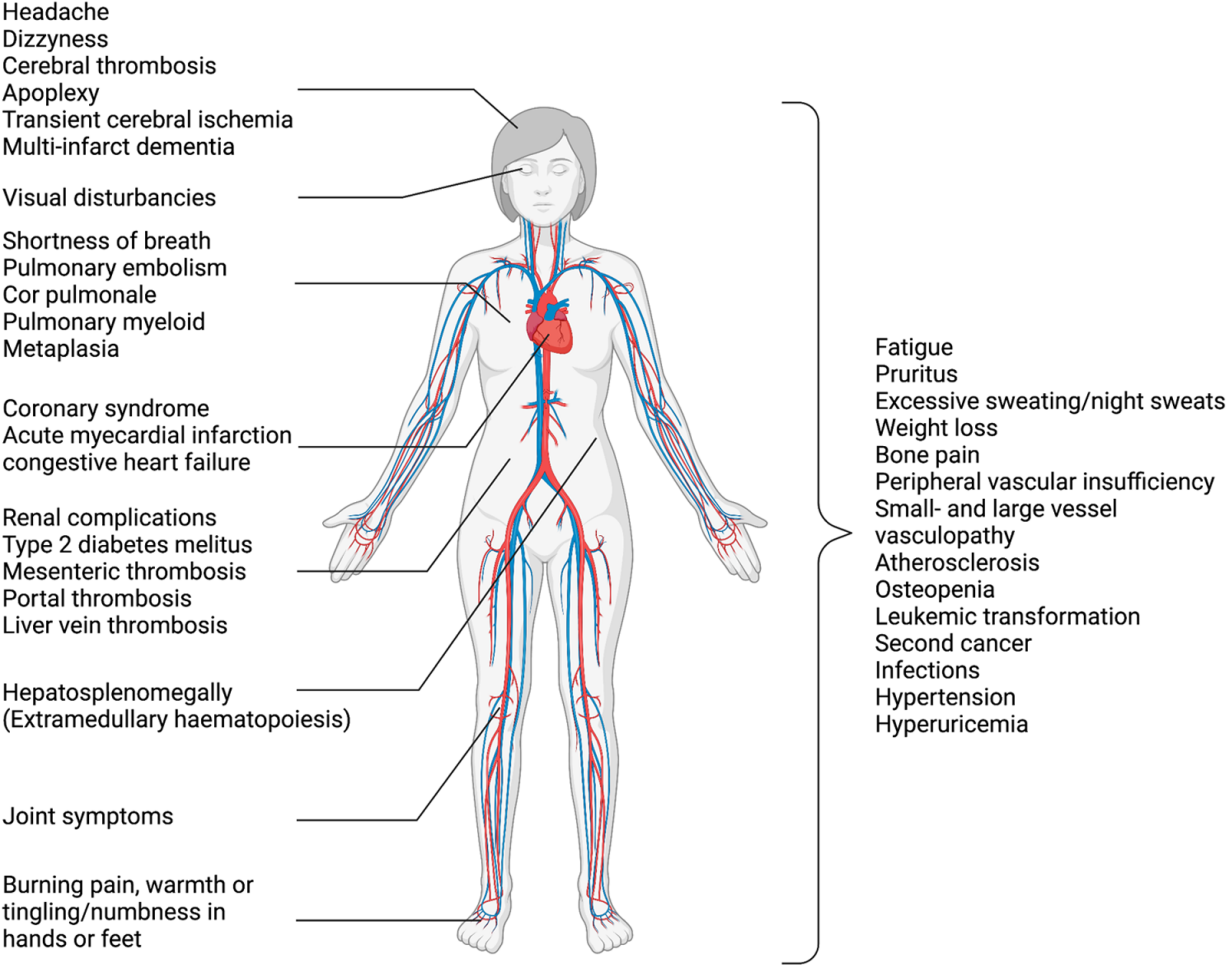
Symptoms and complications in patients with MPNs. Patients with MPNs have a huge symptom and associated disease burden/comorbidity burden, and some of these are presented in this figure. For ocular manifestations in these patients please see review by Liisborg et al. (2020).*Created with BioRender*.

The MPNs often go unnoticed for several years before diagnosis. Unfortunately, the haematological disturbances present in these patients often result in the many complications seen (Figure 4), with possible devastating and debilitating consequences for the person afflicted. These complications are often the primary reason for patients contacting the health care system and, therefore, the reason the diseases are being diagnosed. Cardiovascular diseases, thromboembolic and haemorrhagic events, infections, leukemic transformation to acute myeloid leukaemia (AML) and second cancer are responsible for the increased morbidity and mortality in these patients (Spivak, 2017, Tefferi & Pardanani, 2015).

##### Epidemiology

It is difficult to report incidence and prevalence rates on MPNs, and data are possibly prone to under‐reporting because of the disease's indolent nature. As mentioned, many cases of MPNs go unnoticed for several years and are therefore subjected to delayed reporting. Additionally, differences are seen between countries. A meta‐analysis in 2020 reported a pooled annual incidence of 1.03 per 100,000 for ET, 0.84 per 100,000 for PV, and 0.47 per 100,000 for PMF. The combined pooled annual incidence of the three classic MPNs was reported as 2.58 per 100,000 (McMullin & Anderson, 2020). Prevalence rates are higher since many patients live with the diseases for many years. Prevalence of PV ranged from 0.49‐ 46.88 per 100,000, for ET from 11.00‐42.51 per 100,000, and for PMF from 1.76‐4.05 per 100,000 (only a few studies in the meta‐analysis were available on prevalence). Incidence and prevalence of MPNs in Denmark are shown in Table 4.

**TABLE 4:** Incidence and prevalence of MPNs in Denmark

**MPNs in Denmark** In Denmark, with a population of 5.8 million people, it is estimated that the incidence of the classic MPNs is 350 new cases per year (150 PV, ET 150, PMF 50), corresponding to 6.03 cases per 100.000 per year. (‘Danish National Chronic Myeloid Neoplasia Study Group, 2015. Danish.’ n.d.) A study in Denmark suggests an underestimation of the prevalence of the MPNs since a population‐based screening of 19 958 adult citizens revealed a higher prevalence of the JAK2 mutation in this group than the number of diagnosed patients with MPNs. This raises a concern of an MPN underdiagnosis in the population and an estimated prevalence of 14,000 patients, of which 10,000 are undiagnosed (Cordua et al., 2019).

The median age of diagnosis ranges from 60‐70 years depending on subtype, with PMF patients being older at diagnosis. Again differences are seen between countries (Shallis et al., 2020).

The median survival estimates of ET, PV and PMF patients are reported as approximately 20, 14 and, six years, respectively, worse than age‐ and sex‐matched control populations (Shallis et al., 2020). In patients younger than 60 years at diagnosis, the survival estimates are better with the numbers 33, 24 and 15 years for ET, PV and PMF, respectively (Tefferi & Pardanani, 2015).

Despite the discovery of the three classic acquired driver mutations *JAK2*, *CALR*, and *MPL*, the pathogenesis of MPNs is not fully understood. The driver mutations are considered secondary events, and the initiating event remains to be established. Tobacco use and a history of autoimmune diseases are associated with an increased risk of MPNs. Inherited genetic abnormalities are suspected to increase the risk of acquiring the driver mutations. This is supported by observations of first‐degree relatives having a higher risk of developing MPNs (Shallis et al., 2021). The MPNs are more common in men than women. The overall male:female ratio is 1.2‐1.7. In ET, though, there is a higher incidence in females, male:female ratio 0.5‐0.7. Differences in ethnic groups are seen as well. Inflammation has become recognised as having a crucial role in the development and progression of the MPNs (Hasselbalch, 2012, 2013; Mendez Luque et al., 2019). Inflammation in patients with MPNs will be further described in the “Inflammation” section on below.

##### Treatment

The guideline for the treatment of MPNs is based upon a risk stratification, including age, symptoms and the MPN subtype and threshold values in blood cell counts. The approach has been watchful waiting or only aspirin to prevent thrombotic events in mild cases. Further, phlebotomy is used to treat high levels of red blood cells, or in more severe cases, cytoreductive treatment (Hydroxyurea, Interferon‐alpha, Anagrelide) are used. With advanced disease or the burned‐out phase where the bone marrow cannot produce enough cells, supportive care is used, such as Erythropoietin treatment or transfusions and antibiotics to prevent and fight infection. In some cases, bone marrow transplantation is used. Another drug used to treat myelofibrosis and treatment‐resistant PV is Ruxolitinib, an inhibitor of Janus‐associated kinases (JAK1 and JAK2) (Kim et al., 2021).

However, emerging evidence has set the scene for an early treatment intervention to dampen the massive inflammation and the gradually increasing allele burden seen in these patients, which will also be described in the section “Inflammation” (page 13). Combination therapy with interferon‐alpha, which can reduce the elevated blood cell counts, splenomegaly and the *JAK2* allele burden together with Ruxolitinib, a potent anti‐inflammatory agent, has shown promising results (Hasselbalch & Holmström, 2019; Mikkelsen et al., 2018).

#### AMD MPN connection

Chronic inflammation is a characteristic feature and is believed to play a crucial role in the development and progression of the MPNs (Hasselbalch, 2013, Mendez Luque et al., 2019). Chronic inflammation is also the common link between many prevalent diseases such as type II diabetes, atherosclerosis and cancer (Balkwill & Mantovani, 2001; Ehlers & Kaufmann, 2010). The signalling pathways JAK‐STAT and NF‐κβ, involved in many processes in the body, including immunity, cell division and tumour formation, are also involved in MPNs and are constitutively activated as a consequence of the driver mutations (*JAK2V617F*) (Spivak, 2017).

The chronic inflammation and constitutively activated signalling pathways make the MPNs unique as model diseases for investigating links between chronic inflammation and other conditions. Recently, for example, the MPNs have been proposed as a “Human neuroinflammation model” for the development of Alzheimer's disease (Hasselbalch et al., 2020).

The MPNs have also been associated with various other conditions and diseases, in which inflammation plays a crucial role. Examples are atherosclerosis (Hasselbalch, 2012, 2015), cardiac disease (Reisner et al., 1992), several autoimmune diseases (Barcellini et al., 2013; Barosi, 2014; Kristinsson et al., 2010), chronic kidney disease (Christensen et al., 2014), inflammatory bowel disease (Bak et al., 2020), and increased risk of second cancer (Frederiksen et al., 2011).

A register study from 2017 found an increased risk of AMD in patients with MPNs (Bak et al., 2017), and a pilot study has shown an increased prevalence of drusen in these patients (unpublished results). A common link could again be chronic inflammation, and studying patients with MPNs could tell us more about the role of systemic inflammation in AMD. Other apparent similarities between AMD and MPNs are neoangiogenesis, and the diseases develop primarily in older individuals. In the following three sections, these three factors/characteristics (inflammation, ageing and angiogenesis) observed in both AMD and MPNs will be described, giving an overview of the background knowledge and evidence underlying the objectives and hypotheses of this study.

#### Inflammation

Inflammation is the immune system's complex response to harmful stimuli (injury, pathogens, irritants), and it plays an essential part in fighting pathogens and in healing and repair. Inflammation is a protective response but can become dysregulated and detrimental (Chen et al., 2018; Medzhitov, 2008). Inflammation can be divided into acute‐ and chronic inflammation. The acute response is the one that occurs with tissue injury or infection (showing the classical signs calor (heat), dolor (pain), rubor (redness), and tumor (swelling)). The innate immune system cells are activated through their pattern‐recognition receptors (PRRs), sensing pathogen‐associated molecular patterns (PAMPs)‐ and damage‐associated molecular pattern molecules (DAMPs). This activation results in the secretion of cytokines and chemokines; among these are IL‐1β, IL‐6, IL‐8, IL‐12 tumour necrosis factor (TNF)‐α and many more (Kauppinen et al., 2016; Lentsch & Ward, 2000), which again affect numerous cells. For example, one effect of IL‐1β and TNF‐α is the activation of endothelial cells, with increased vascular permeability, vasodilation, expression of leukocyte adhesion molecules and cytokines, driving an increased movement of cells, fluids, and plasma proteins from the blood to the injured tissue. The endothelial cells phenotype also changes from antithrombotic to prothrombotic (Blann, 2000). An example of the effect of IL‐8 is the attraction of neutrophils. Leukocytes reach their target site (inflammation site) by following a chemokine gradient (Kauppinen et al., 2016, Lentsch & Ward, 2000). The acute response ceases within days (Germolec et al., 2018; Medzhitov, 2008).

Persistent inflammation, either due to prolonged stimulation/exposure or an inappropriate response/adaptation from the host, can lead to chronic inflammation, often referred to as slow and long‐term with subtler symptoms. A shift from dominantly activated granulocytes and platelets in acute inflammation to recruitment and activation of more mononuclear cells are seen, with the following risk of tissue damage and fibrosis (Germolec et al., 2018). Numerous diseases such as atherosclerosis (Lathe et al., 2014; Stoll & Bendszus, 2006), type‐2 diabetes (Calle & Fernandez, 2012), obesity (Ellulu et al., 2017), and almost all age‐related degenerative diseases, including AMD (McGeer & McGeer, 2004), have been associated with chronic inflammation, and it is a hot and very debated topic. With age, individuals also tend to develop a non‐specific low‐grade inflammatory state termed “inflamm‐ageing”, which will be described further in the next section on page 15.

**FIGURE 5:**
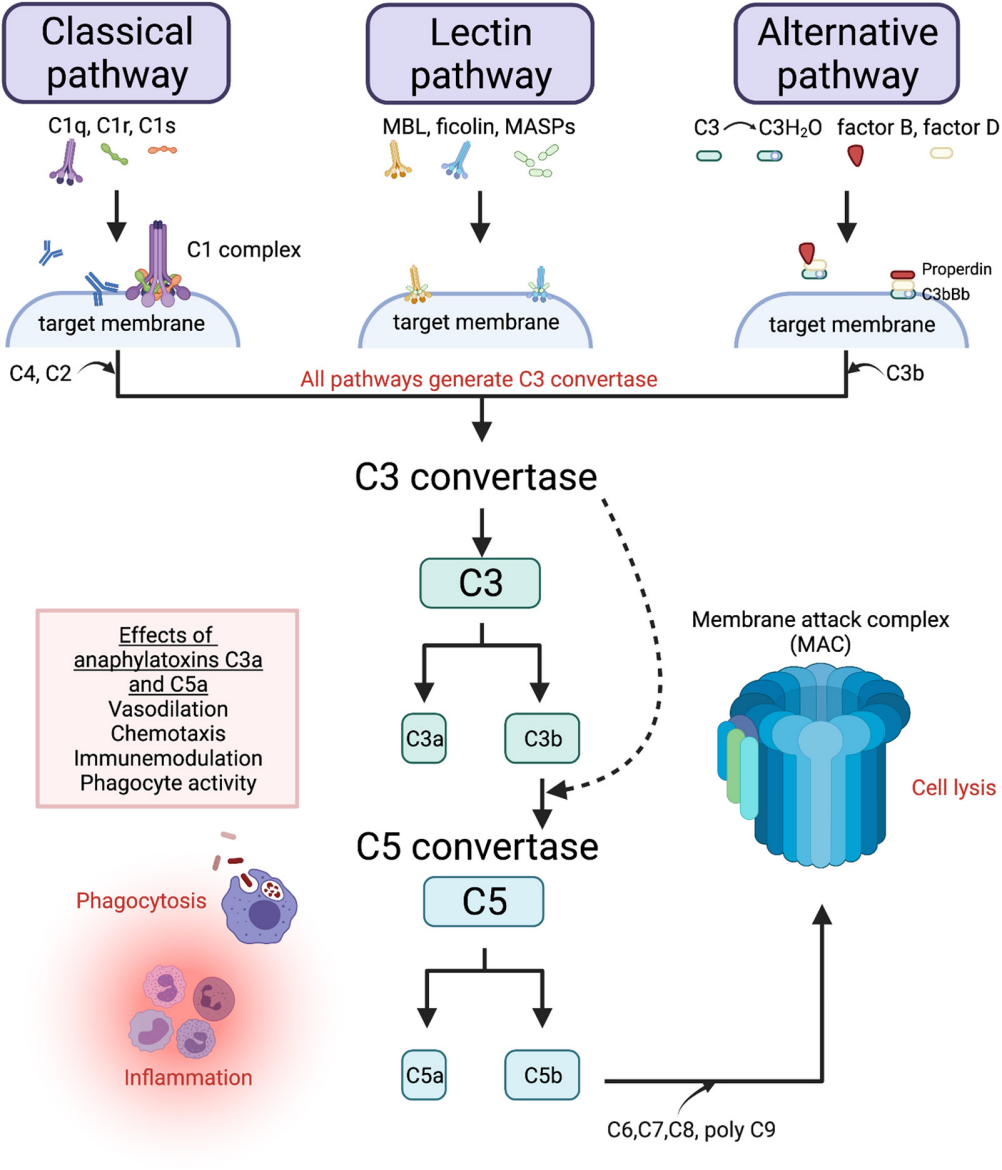
A simplified drawing of the complement system. The three different complement activation pathways culminate in a common terminal pathway. The classical pathway is activated by antigen/antibody complexes (C1q interact with C1r and C1s to form the C1 complex that interacts with antigen/antibodies on the pathogen surface). The proteases C1r and C1s cleave C2 and C4, generating the classical pathway convertase C4b2a (not shown). The lectin pathway is initiated by mannose‐binding lectin (MBL) or ficolin binding to carbohydrates on the pathogen or target membrane. The MBL‐associated serine proteases (MASPs) then cleave C2 and C4, generating the C4b2a convertase (not shown). In the alternative pathway, constant low‐level hydrolysis of C3 to C3H_2_O is seen, augmented by factors B, D and Properdin. The pathway functions as an amplification loop when C3b bind to a target membrane (pathogen, damaged tissue, foreign material) and interact with the C3H_2_0, factors B and D to form the alternative pathway convertase C3bBb stabilised by Properdin. All three pathways converge in the final pathway where C3 convertases cleave C3 forming C3a and C3b. C3b bind to C3 convertases generating C5 convertases. The C5 convertase cleaves C5 into C5a and C5b. The anaphylatoxins C3a and C5a activate and attract inflammatory cells and lead to vasodilation, inflammation, and phagocytosis. C5b bind to C6, C7, C8 and multiple C9, forming the membrane attack complex (MAC). The MAC forms pores on target membranes and can cause cell lysis. *Figure created with BioRender*.

The complement system regulates various steps of the inflammatory response, and it is an essential part of the innate immune system, constituting the first line of defence against infections. However, it also has other functions as an immunoregulatory system playing a part in adaptive immunity. Complement are proteins present in the body fluids (soluble and membrane‐bound). The “inactive” plasma complement proteins circulate in the bloodstream and can be activated in several ways and interact to activate different complement pathways. The pathways include the classical (antigen‐activated) pathway, the alternative pathway (can be activated by a pathogen alone) and the lectin pathway (activated by lectin‐type proteins). The three pathways all lead to C3 convertase activation with the creation of C3a and C3b, resulting in different effector activities: inflammation, phagocytosis and formation of the membrane attack complex (MAC) (Figure 5) (Ling & Murali, 2019). A complement system also exists in the retina where RPE and microglial cells are the primary producers of complement components (Anderson et al., 2010).

##### Inflammation in MPN and AMD

**FIGURE 6:**
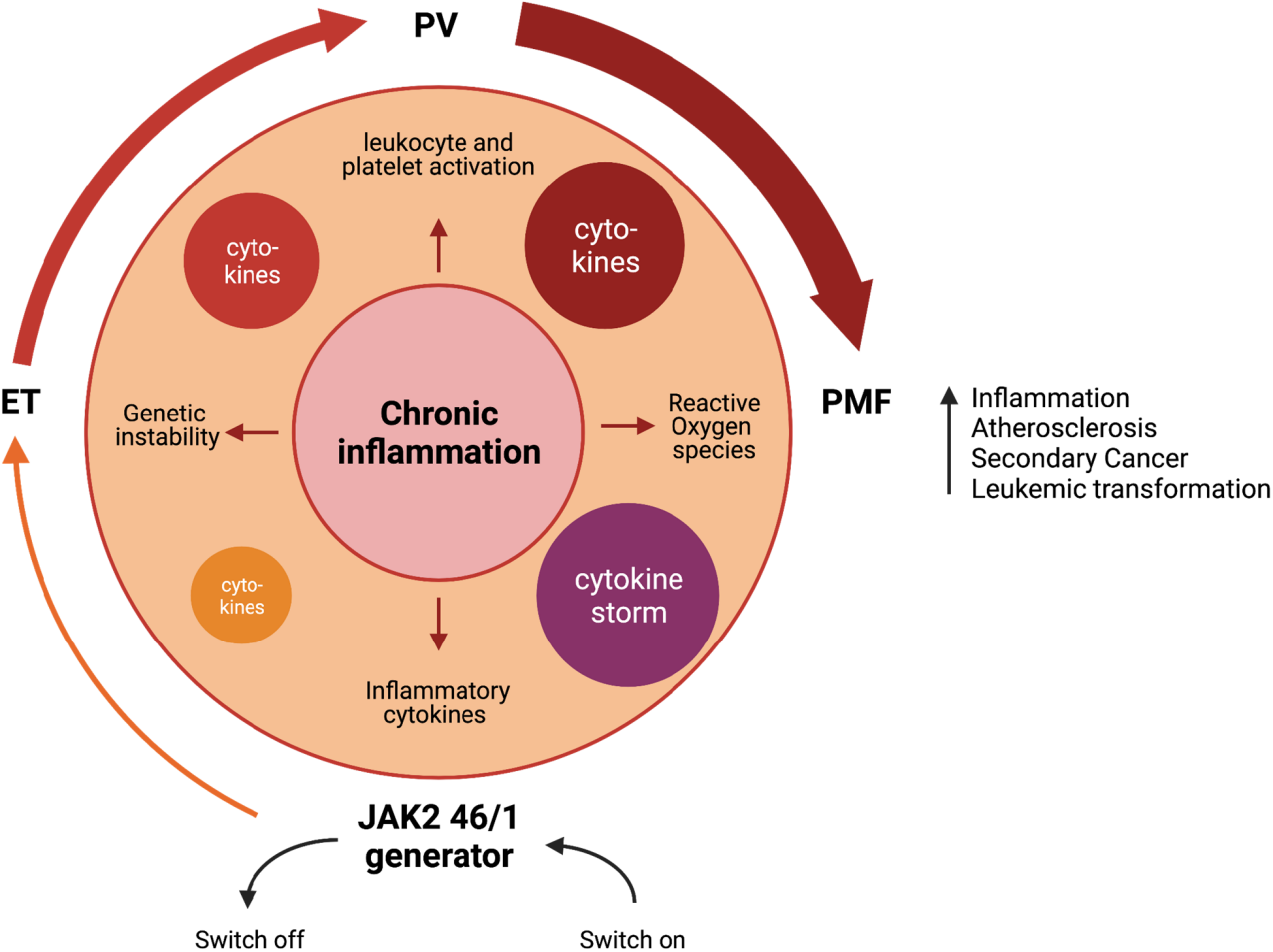
Redrawing of figure “MPN Human Inflammation Model” (Modified and simplified) from Hasselbalch HC, Bjørn ME. MPNs as Inflammatory Diseases: The Evidence, Consequences, and Perspectives. *Mediators Inflamm* 2015; 2015: 1–16.^67^ Chronic inflammation initiate and drives clonal expansion and is represented by an increased level of inflammatory cytokines and reactive oxygen species (ROS). This creates a dangerous microenvironment with genetic instability – DNA damage. When the JAK2 mutation happens, it increases the cytokine and ROS levels further and raises platelet and or leukocyte levels giving rise to even more inflammatory products. This creates a self‐fuelling positive feedback loop – a vicious cycle of increasing inflammation and clonal expansion. The rounded arrows outside the large circle represents both the mutational burden (allele burden), the inflammation and comorbidity burden in the different stages in the biological continuum of the MPNs from early cancer state ET/PV to the late burned‐out phase of myelofibrosis with increased risk of leukemic transformation. The coloured smaller circles represent the increasing levels of inflammatory mediators (cytokines) also following the biological continuum. JAK2 46/1: the JAK2 46/1 haplotype. ET: Essential thrombocythemia, PV: Polycythaemia vera, PMF: primary myelofibrosis. *Created with BioRender.*

It is well accepted that there is a causal link between chronic inflammation and cancer (Colotta et al., 2009; Coussens & Werb, 2002; DiDonato et al., 2012; Fan et al., 2013; Karin & Greten, 2005). MPNs have also become increasingly recognised as inflammatory diseases. The diseases are characterised by hyperactivation of the JAK‐STAT and NF‐kβ pathways (Fisher et al., 2021; Hasselbalch, 2013; Mendez Luque et al., 2019; Netea et al., 2017), and elevated levels of inflammatory cytokines (e.g. IL‐4, IL‐6, IL‐8, IL‐11, TNF‐α and many growth factors)(Hasselbalch & Bjørn, 2015) and a following chronic inflammatory state. The cytokines correlate with disease initiation and progression and the symptom burden and prognosis (Hoermann et al., 2015). A “Human Inflammation Model” for cancer development in the MPNs has been proposed (Andersen et al., 2017; Hasselbalch, 2013). Here, inflammation is suggested to initiate and is a driving force of clonal evolution in MPNs. Chronic inflammation causes an environment with continuous release of inflammation products and elevated reactive oxygen species (ROS) levels with the following risk of DNA damage. This genomic instability could give rise to the mutations seen in MPNs, resulting in rising levels of activated leukocytes and/or platelets and an even further release of inflammatory products creating, again, a higher risk of mutagenesis. Thus, a positive feedback loop has been created ‐ a self‐fuelling vicious cycle driving clonal expansion (Figure 6). This model supports the contention of early therapeutic intervention with treatment dampening the chronic inflammation when the tumour burden is minimal (statins, JAK inhibitors), but also agents impairing clonal evolution (interferons) (Hasselbalch & Bjørn, 2015; Tefferi & Pardanani, 2015).

Inflammation can emerge at three levels: a tissue cell (autonomous) response, a local tissue's immune system response, or a systemic immune system response (Chen & Xu, 2015). In AMD, it is widely accepted that local inflammation (tissue level) plays a role in the pathogenesis (Ambati et al., 2013; Anderson et al., 2002, 2010; Chen & Xu, 2015; Kauppinen et al., 2016; Kersten et al., 2018). This response (sometimes also referred to as para‐inflammation) is thought to be “protective” and poorly understood why it becomes detrimental. It is known, though, that ageing involves an accumulation of oxidative stress, and oxidative damage are believed to be the initial trigger of AMD (Chen & Xu, 2015). When many cells of the tissues in the eye are stressed, and or insults persist for longer periods, as is the case with ageing, the cell‐autonomous response of tissue cells (primarily RPE) to repair the damage is not enough to overcome the stress and return to normal. This activates the local immune regulatory system orchestrated by the RPE cells, microglia/macrophages, and other retinal cells. Again if the stress exceeds the repair mechanisms of the local immune response, these cells may secrete cytokines and chemokines to activate and attract cells from the systemic immune system, such as neutrophils and monocytes (which turn into macrophages) (Chen & Xu, 2015; Medzhitov, 2008). The retina is an immune‐privileged tissue protected by the BRB and the local immune regulatory system consisting of retinal innate immune cells and the local complement system. However, animal studies and donor eyes from patients with GA have revealed that the BRB does not seem to be an obstacle for circulating cells to enter the retina (Guillonneau et al., 2017; Sennlaub et al., 2013).

In addition to the local inflammation, indications of a systemic contribution are emerging. Previous studies have, for instance, shown increased plasma concentration of complement fragments (Ebrahimi et al., 2013; Machalińska et al., 2009; Reynolds et al., 2009; Scholl et al., 2008; Sivaprasad et al., 2007), decreased levels of complement regulatory proteins (Singh et al., 2012), increased activations of the JAK2/STAT3 (Chen et al., 2016) and NF‐kβ pathways (Kauppinen et al., 2016), increased serum levels of CRP (Hong et al., 2011; Kikuchi et al., 2007; Mitta et al., 2013; Seddon et al., 2004), and various inflammatory cytokines, such as IL‐1β, IL‐4, IL‐8, IL‐17, TNF‐α (Liukkonen et al., 2022; Nassar et al., 2015) and a higher neutrophil‐to‐lymphocyte ratio (NLR) (Niazi et al., 2019). Moreover, ageing is associated with inflamm‐ageing (described in the next section), and AMD is an age‐related disease.

In summary, both patients with MPN and AMD share hyperactivation of the JAK‐STAT and NF‐kβ pathways and the rise in cytokine levels.

#### Ageing

Ageing is the process of growing older and is often associated with a decline of function over time in physical and mental capacity and an increased risk of morbidity and mortality. Ageing from a biological perspective is the accumulation of changes and damage over time, and all organ systems are affected. Changes occur in all human beings but are only loosely associated with a person's chronological age, which does not always match the biological age. The impact of and differences in genetic, environmental, demographical, and behavioural factors results in a great variety between individuals. The health outcomes of individuals reaching old age are not only dependent on the accumulation of damage but also the capacity of the individual's physiological systems to adapt to damage and the capacity to retain body functions (Christensen et al., 2008; López‐Otín et al., 2013; Vallejo et al., 2011; van Beek et al., 2016). López‐Otín et al. (2013) have defined nine hallmarks of ageing, which describe the common denominators of ageing (Stem cell exhaustion, altered intercellular communication, genomic instability, telomere attrition, epigenetic alterations, loss of proteostasis, deregulated nutrient sensing, mitochondrial dysfunction and cellular senescence). Ageing is a contributing risk factor for many diseases. As we grow older, illness and disease occur more frequently, especially neurodegenerative‐, cardiovascular diseases and cancer (Hou et al., 2019; Niccoli & Partridge, 2012). Ageing is the greatest risk factor of developing AMD (Chakravarthy et al., 2010; Lambert et al., 2016). Myeloproliferative neoplasms are typically diagnosed later in life (Tefferi et al., 2013).

In this study, we focus on the impact of age on the immune system. Immunosenescence is a term used for age‐related changes in the immune system, both in the innate and adaptive parts. Often the term is associated with declining immune system functions, and some functions are indeed decreased, but others are increased (Fulop et al., 2018; Goronzy et al., 2015; Moro‐García et al., 2013). In general, older adults have less robust immune responses than younger individuals (Vallejo et al., 2011). Another term used for changes in the immune system is Inflamm‐ageing, a tendency for individuals to develop a non‐specific low‐grade inflammatory state defined by an increase in pro‐inflammatory biomarkers or mediators with age (Bektas et al., 2017). Chronic inflammation is, as ageing, a strong risk factor for the development and progression of many diseases such as the mentioned neurodegenerative‐ and cardiovascular diseases and cancer.

A consistently observed age‐related change of the adaptive immune system is the decrease in the CD8+ naïve T cell population and the increase in CD8+ memory T cells (Pawelec, 2017). The CD4+ and CD8+ T cell compartments can phenotypically be divided into four distinct subpopulations: Naïve, central memory, effector memory and effector memory CD45ra positive T cells (Fulop et al., 2018). When naïve T cells are activated, they differentiate into central memory or effector memory cells, and the effector cells can be regarded as senescent T cells, which, therefore, accumulate with age (Callender et al., 2018; Pereira et al., 2020). T cells originate from the thymus and thymic output peaks at puberty with a following decline. Ultimately involution of the thymus is seen. Hereafter, T cells are maintained and regenerated from the existing peripheral T cell pool, and principles of stem‐cell exhaustion apply to naïve T cell ageing leading to the age‐dependent decline in naïve T cells (Goronzy et al., 2015).

Other changes in the T cell compartment are the age‐dependent increase in CD56 expression in both CD4+ and CD8+ T cells (Lemster et al., 2008; Vallejo et al., 2011) and the loss of CD27 and CD28 expression.

CD56 is a marker of natural killer (NK) cells. The function of CD56 expression in NK cells remains unanswered, but upregulation of expression seems to be related to the degree of activation (Van Acker et al., 2017). A CD56^−^ subpopulation can exist. However, these cells are commonly found in pathologic conditions such as HIV, chronic hepatitis and autoimmune disease and these NK cells are reported to have impaired or dysfunctional cytokine production and cytolytic capacity (Van Acker et al., 2017). Evidence also suggests that CD56^−^ NK cells increase with age (Campos et al., 2014). The CD56 expression on T cells is also associated with effector functions, and these cells can exert activity resembling NK‐cell killing in an inflammatory milieu (Chan et al., 2013; Pittet et al., 2000).

CD28 is a co‐stimulatory receptor responsible for T cell activation, proliferation and survival (Weng et al., 2009). T cells losing CD28 exhibit features such as reduced T cell receptor diversity, deficiency in antigen‐promoted proliferation and enhanced cytotoxicity and suppressive functions (Weng et al., 2009). CD27 is a member of the TNF receptor family and a co‐stimulatory molecule and has, as CD28, been used to study differentiation of T‐cells (Fritsch et al., 2005).

Inflamm‐ageing and T cell ageing are among the most well‐characterised age‐related changes of the immune system, and both have been associated with AMD (Chen & Xu, 2015; Faber et al., 2013; Subhi et al., 2017). For example, one previous study found that patients with neovascular AMD had more CD56+ cells in the CD8+ T cell compartment and more loss of the co‐stimulatory markers CD28 and CD27 compared to healthy individuals, indicating accelerated T cell ageing (Subhi et al., 2017). Other studies have found elevated serum and plasma levels of inflammatory cytokines (Liukkonen et al., 2022, Nassar et al., 2015), complement fragments and CRP (Hong et al., 2011; Mitta et al., 2013). To our knowledge, no studies have evaluated T cell CD56 expression in patients with MPNs, but these patients also have elevated levels of inflammatory cytokines (Fisher et al., 2021; Hasselbalch & Bjørn, 2015).

#### Angiogenesis

Angiogenesis is defined as the formation of new blood vessels from pre‐existing ones (Risau, 1997), and it is a physiological process during embryonic development, female reproductive cycling, and wound healing. Pathological angiogenesis occurs in cancer and numerous other conditions, including inflammatory diseases. In wound healing and pathological conditions, angiogenesis is accompanied by and requires inflammation. Angiogenic stimuli include tissue ischemia, hypoxia, inflammation, and shear stress (Folkman, 1995).

Regulation of angiogenesis is a complex process involving angiogenic and angiostatic factors, and vascular homeostasis (angiostasis) requires the balance between these activators and inhibitors. Several growth factors are pro‐angiogenic, such as vascular endothelial growth factor (VEGF), and chemokines, which are chemoattractant cytokines, can have both inhibitory and stimulatory properties on angiogenesis. Cytokines and chemokines accumulate at sites of inflammation, exerting their effect on endothelial cells but also by attracting immune cells (e.g. monocytes and lymphocytes), which may, in turn, secrete pro‐ and anti‐angiogenic factors, thereby making inflammation and angiogenesis coupled processes (Romagnani et al., 2004). Growth factors also stimulate endothelial cells to proliferate and migrate towards a growth factor gradient (Dimberg, 2010). Many chemokines bind multiple chemokine receptors adding to the chemokine systems complexity. Also, chemokines and their receptors cross‐talk with pro‐angiogenic growth factors such as VEGF and fibroblast growth factor, further adding complexity.

Chemokines are grouped according to their amino acid composition and the position of their N‐terminal cysteine residues. Chemokines are divided into four subfamilies, the CC, CXC, CX3C and XC and are for nomenclature, followed by “L” and a number. The “C” represents cysteine and “X” any amino acid. The chemokines exert their properties on receptors named after the same nomenclature with “R” instead of “L” (Hughes & Nibbs, 2018; Luster, 1998). The CXC chemokines are divided into two subcategories based on the presence of a particular amino acid sequence (Glu‐Leu‐Arg) near the amino‐terminal end critical for binding and activity of the chemokine ‐ the so‐called ELR motif. Chemokines with the ELR motif are generally potent promotors of angiogenesis, while chemokines lacking the ELR motif are inhibitors (Balestrieri et al., 2008).

Angiogenesis is a central event in AMD and MPNs, in the retina and bone marrow, respectively, with VEGF as the most important pro‐angiogenic agent (Fleckenstein et al., 2021; Hasselbalch, 2012; Medinger et al., 2009; Medinger & Passweg, 2014). The interferon (IFN)‐γ inducible chemokines are ELR negative and thus angiogenesis inhibitors. They all exert their effect through the chemokine receptor CXCR3 (Groom & Luster, 2011). We have found the CXCR3 receptor to be lower in patients with nAMD compared to healthy controls (Falk et al., 2014; Singh et al., 2017). A Turkish study from 2021 have found reduced expression of CXCR3 in peripheral blood mononuclear cells (PBMC) obtained from patients with PV, but only the abstract is in English. To our knowledge, no other studies have reported CXCR3 expression on T cells from patients with MPNs. The CXCR3 receptor is also involved in T cell differentiation, thereby also coupling angiogenesis and ageing, as well as angiogenesis and inflammation mentioned earlier. Further, as described in the inflammation and ageing sections, these two factors are also interconnected. Therefore, alterations in inflammation, ageing and angiogenesis mutually affect each other.

### OBJECTIVES AND HYPOTHESES

As described previously, AMD is a multifactorial disease with a complex interplay between demographic and environmental factors and genetic susceptibility. The exact aetiology or how these factors cause macular damage are not fully understood. A great deal of focus in AMD research has been on local changes in the eye, but also evidence of alterations in the systemic circulation has emerged, and some changes acknowledged as being a part of the pathogenesis. Especially associations between chronic inflammation and AMD have been documented. Some of the other identified systemic alterations are pertaining to the complement system, ageing T cells, and the chemokine system.

The MPNs are characterised by neoangiogenesis and fibrosis in the bone marrow and massive systemic inflammation. As described earlier, these diseases have been used as model diseases for investigating links between chronic inflammation and other conditions. Studying patients with MPNs is likewise a unique possibility to investigate the role of systemic inflammation in AMD development. The similarities between AMD and MPNs are also evident: inflammation, angiogenesis and fibrosis, and the diseases develop primarily in the elderly.

We know from the register study by Bak et al., 2020 that patients with MPNs have a higher risk of AMD. We speculate that this increased risk is due to these patients' massive chronic inflammatory state. Therefore, the objectives of this project are:

1. To investigate if patients with MPNs show a higher prevalence of retinal changes compatible with AMD and from an earlier age. **(Study I, Paper I).** *Our null hypothesis is that patients with MPN do not differ in prevalence of drusen and AMD compared to population estimates*.
2. To assess the immunological similarities in patients with MPNs and AMD ‐ to identify previously relevant changes in the immune system found in AMD, which are shared with MPNs. We hypothesise that the following triad contributes to the development of nAMD: inflammation, ageing and angiogenesis. These factors are interconnected and affect each other and will be studied in the systemic circulation of the included patients. More importantly, if patients with MPN and drusen show overlap with patients with AMD in these immunological signatures and are different from patients with MPNs and normal retinas, this could support that these specific changes could be a part of the drusen/AMD pathogenesis. With this, we hope to identify an immunological profile of “high‐risk drusen individuals”. **(Study II).** Study II was further divided into three immunological substudies: study II‐a ‐ inflammation **(Paper‐II)**, study II‐b – ageing **(Paper III)** and study II‐c – angiogenesis **(Paper IV)**. *Our null hypothesis is that MPNd and MPNn do not differ in the systemic markers investigated*.

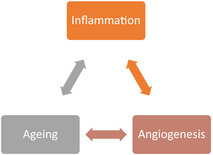


#### Biomarkers and models

Research has tried to obtain reliable biomarkers for AMD, but none have been found to detect early AMD. Suppose we can find changes at the early or intermediate level, which increase the risk of developing late AMD, then we can learn more about the mechanisms of AMD pathogenesis, detect the disease, have a prognosis earlier, and potentially develop new therapies as well as monitor the disease response to therapy (Lambert et al., 2016). The MPNs could potentially be a model of drusen development.

## METHODS

### STUDY DESIGN & ETHICS

All the studies we conducted for this thesis had an observational cross‐sectional design. We carried out the studies at the Department of Ophthalmology at Zealand University Hospital (ZUH) – Roskilde, Denmark. They were all approved by the Ethics Committee in Region Zealand, Denmark (SJ‐588 and SJ‐679), the Danish Data Protection Agency (REG‐015‐2018) and adhered to the tenets stated in the Helsinki Declaration. The participants provided both oral and written informed consent after a thorough explanation of the studies.

### PARTICIPANTS

We included four types of patients for a single visit. From the outpatient program at the Department of Haematology, ZUH, we included patients with MPNs according to the WHO2016 criteria (Barbui et al., 2018), both patients showing signs of AMD (eAMD and iAMD) and patients with healthy‐looking retinas. We included patients with intermediate and neovascular AMD from the outpatient program at the Department of Ophthalmology, ZUH. The patients with AMD were included according to the classification system introduced by the Beckman Initiative for Macular Research Classification Committee (Ferris et al., 2013). Patients with nAMD were diagnosed according to standard procedures and subjected to Early Treatment of Diabetic Retinopathy (ETDRS) evaluation, slit‐lamp bio‐microscopy or fundus photography, OCT and retinal angiography.

For study I, we included 200 patients with MPNs. Of these patients, 35 with AMD signs and 28 with normal healthy retinas were also included in study II along with 29 patients with nAMD and 28 with iAMD. We sampled blood from these patients and subjected them to a questionnaire about their medical history, current medication use, tobacco habits and alcohol consumption. We used height and weight to calculate body mass index (BMI). To avoid disturbances in our immunological measurements, we excluded patients with other concurrent cancer, inflammatory or autoimmune diseases, patients receiving immunomodulating treatment and patients with CRP levels >15 (indicating an ongoing immune reaction). Patients with nAMD who had received anti‐VEGF therapy injection within the last eight weeks (aflibercept) or four weeks (ranibizumab) were also excluded. We included patients between July 2018 and November 2020. For patient demographics, please see table 1 in the included papers (Appendix A page 35).

### OPHTHALMIC EXAMINATIONS

All patients had their pupils dilated with tropicamide 1%. We acquired a fundus photography on every patient for substudy I while only performing OCT on 150. For substudy II, patients had an eye examination with visual acuity testing measured on an ETDRS chart before pupil dilation, and we hereafter obtained a fundus photography, an OCT‐ and a FAF image.

#### Fundus photography and grading

Fundus photography provides a colour image of the retina. The photo is taken through the pupil, preferably after dilation, to allow light to enter the eye resulting in better photo quality.

**FIGURE 7:**
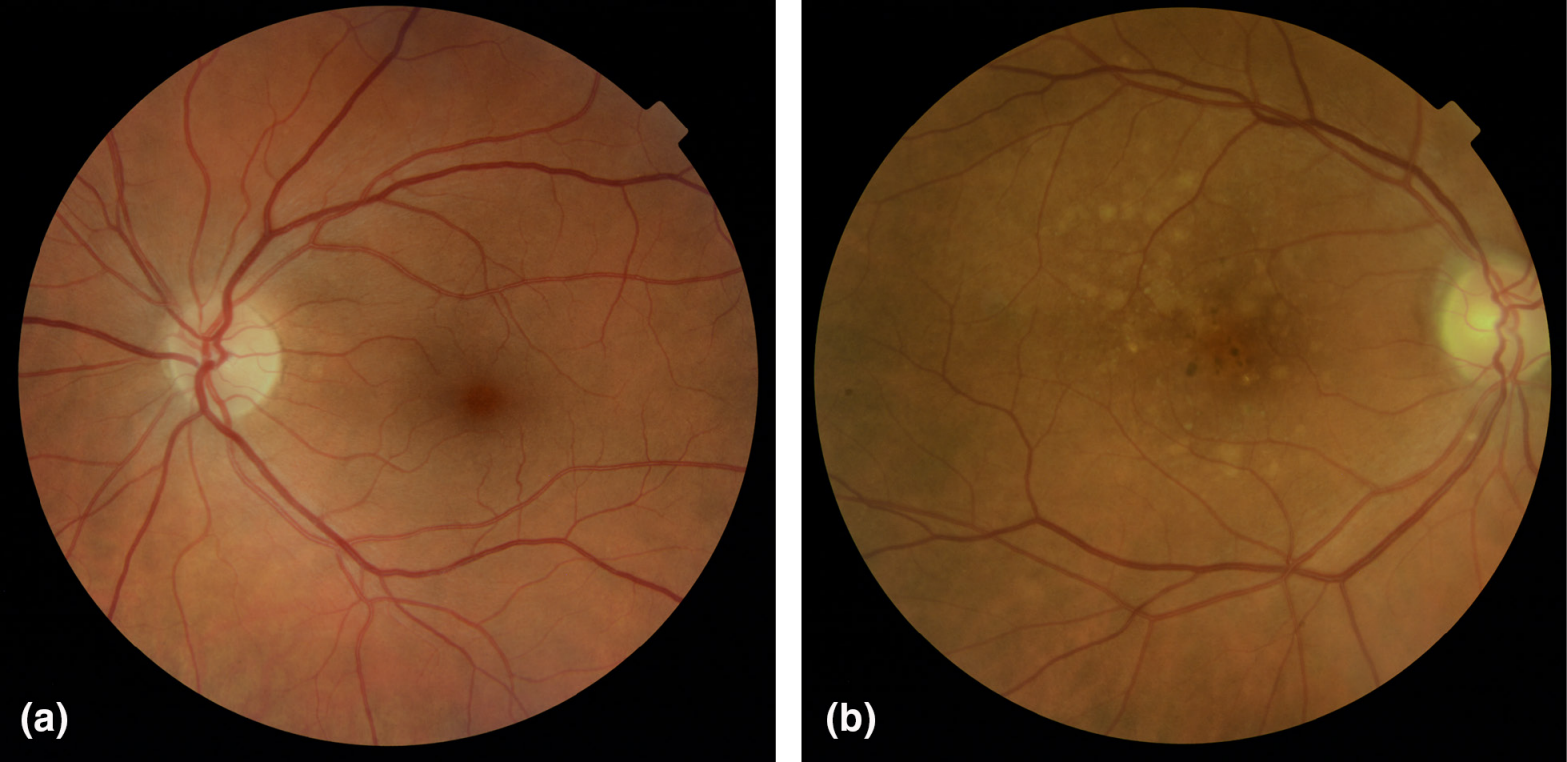
Colour fundus photography of (a) an individual with a healthy retina (b) a patient showing drusen associated with AMD. From IMAGEnet iBase.

On all patients, we obtained stereoscopic 45° colour fundus photographs centred on the macula (model TRG‐NW8, Topcon, Tokyo, Japan) (Figure 7). The photographs were evaluated in IMAGEnet i‐base version 3.25.0 (Topcon, Tokyo, Japan).

In study I, we graded the images using a simplified version of the Wisconsin age‐related maculopathy grading system (WARMGS) (Ferris et al., 2013). This was done to evaluate drusen prevalence, size, area‐covered‐by‐drusen, pigmentary abnormalities, and compare our results with published estimates from three large population studies (The Beaver Dam Eye Study (Klein et al., 1992), The Blue Mountains Eye Study (Mitchell et al., 1995), and The Rotterdam Eye Study (Vingerling et al., 1995)). This method is described in detail in the supplemental material of paper I (page 35). For substudy II, we used the photos to determine AMD status according to the Beckmann classification system (Ferris et al., 2013).

#### Optical coherence tomography and grading

**FIGURE 8:**
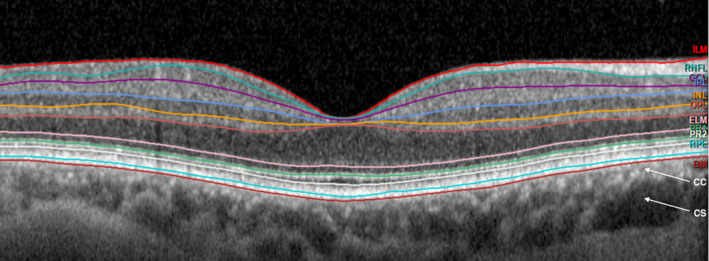
Optical Coherence Tomography (OCT) image showing the layers of the macula of a healthy individual. BM, Bruch's membrane; ELM, external limiting membrane; GCL, ganglion cell layer; ILM, inner limiting membrane; INL, inner nuclear layer; IPL, inner plexiform layer; OPL, outer plexiform layer; PR1/2, photoreceptor inner and outer layer; RNFL, retinal nerve fibre layer; RPE, retinal pigment epithelium. Arrows show choroidea, CC, choriocapillaris; CS, choroidal stroma. The neuroretina is defined as the part of the retina from the INL to the PR. From Heidelberg Eye Explorer. Reprinted from The Lancet, EClinicalMedicine 2020; 26: 100526, Liisborg C, Nielsen MK, Hasselbalch HC, Sørensen TL, Patients with myeloproliferative neoplasms and high levels of systemic inflammation develop age‐related macular degeneration. (supplemental material), Copyright (2020), with permission from Elsevier. https://www.thelancet.com/journals/eclinm/article/PIIS2589‐5370(20)30270‐4/fulltext#articleInformation.

Optical coherence tomography (OCT) is a non‐invasive imaging technique using harmless light waves to obtain high‐resolution cross‐sectional images of the retina (Figure 8). This technique allows us to differentiate and measure retinal layers and thickness. In substudy I, we performed OCT (SD‐OCT, Heidelberg Engineering, Germany) on 150 patients. We hereafter examined the images in Heidelberg Eye Explorer version 1.9.10.0 using the automated segmentation and the thickness profile part of the software to measure the thickness of the retinal layers. This examination is described in further detail in the supplemental material of paper I (page 35)

#### Fundus autofluorescence

**FIGURE 9:**
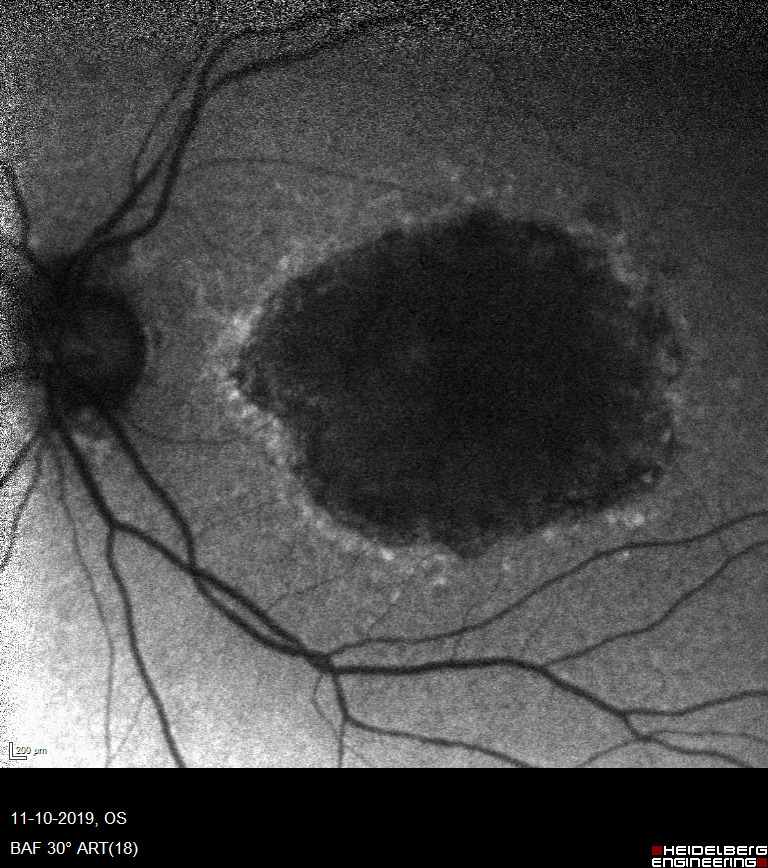
Fundus autofluorescence (FAF) image of a patient eye with geographic atrophy (GA) The image shows a large central atrophic area. *From Heidelberg Eye Explorer*.

Patients in study II also had a fundus autofluorescence (FAF) photo taken. In FAF, the retina is illuminated with blue light that causes certain molecules in the RPE to autofluorescence. This fluorescence is used to create an image of the retina (Figure 9). Areas of RPE loss will be recognised as a decreased signal (darker areas) in the FAF image. In patients with GA, there is RPE loss, and this technique is therefore optimal for recognising areas of atrophy. We used these images to help diagnose or exclude GA in the included patients.

### BLOOD SAMPLES

#### Blood vacutainers and CRP analysis

For substudy II, we sampled venous blood from antecubital veins collected in different tubes from BD Biosciences (Franklin Lakes, NJ, USA). Two tubes with ethylenediaminetetraacetic acid‐coated (EDTA) were used for flow cytometric analyses. One EDTA tube was sent to the Kennedy Centre, Denmark, for storage, and later single‐nucleotide polymorphisms (SNP) analyses at BioXpedia, Denmark. One tube with lithium heparin stabilised blood, hereafter run on Dimension Vista 1500 (Siemens Healthineers, Erlangen, Germany), for plasma CRP analysis. Two tubes with lithium heparin stabilized blood to isolate plasma and two tubes with silica act clot activator to isolate serum by centrifuging. The plasma and serum were immediately stored at ‐80°C and later used for further analyses with immunoassays at the Technical University Denmark (DTU). All blood samples were collected and handled by the same investigator (CL), and the blood for flow cytometry was analysed within 4 h of phlebotomy.

### Flow cytometry

**FIGURE 10:**
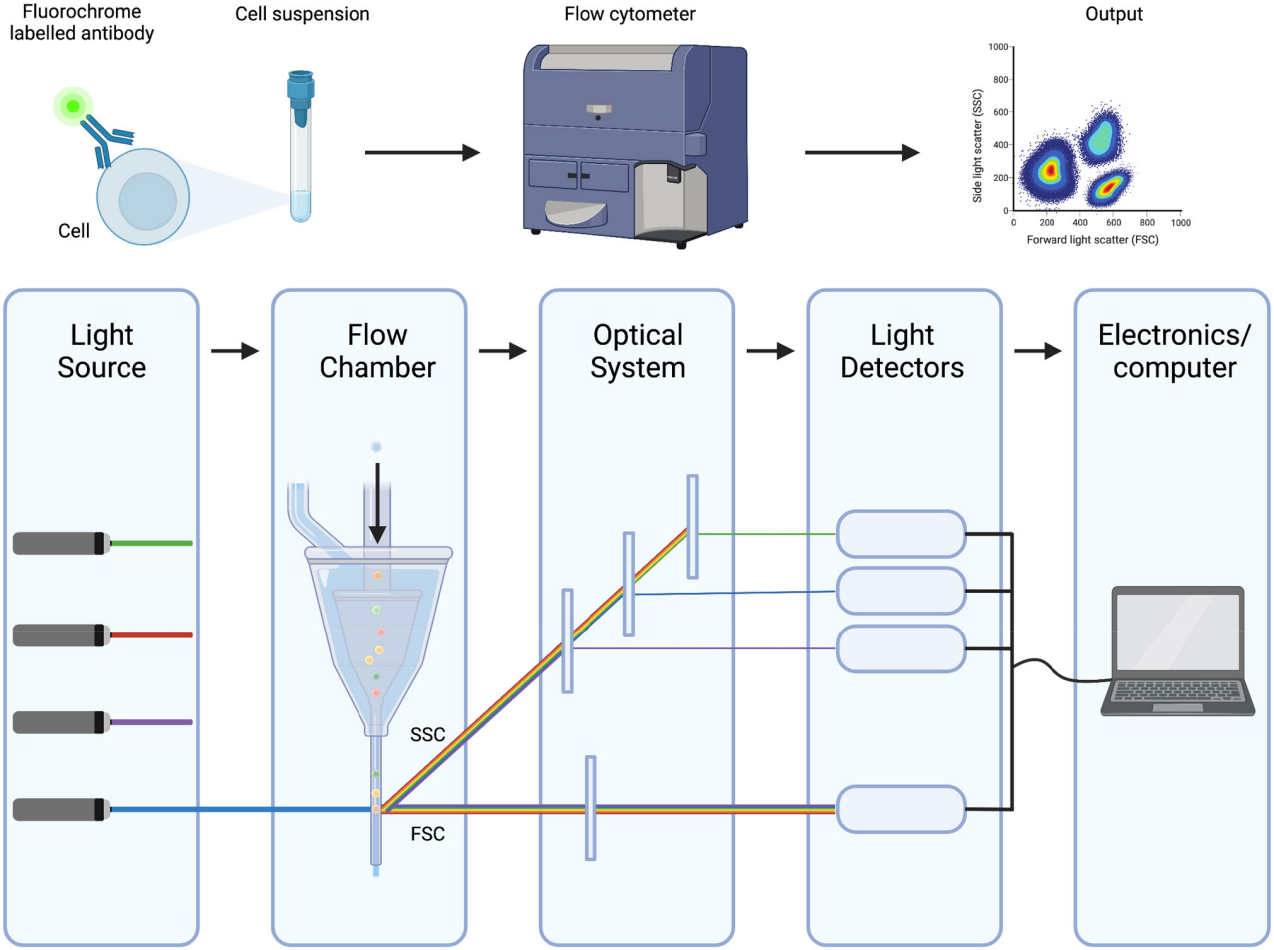
Basic components of a flow cytometer – simplified. The cell suspension contains cells with fluorochrome labelled antibodies. The suspension is run on a flow cytometer which contains the above five basic components marked with blue boxes. The **flow chamber**/fluidics system funnels the suspension through a nozzle that forges a stream of single cells. The cells flow past a set of focused lasers lasers (**light source**). When the light hits the cells, it is scattered. The scattered light is detected by two types of optical detectors (the **optical system**). One detects light along the light path of the laser, referred to as forward scatter (FSC) and the other measures scatter at other angles, the side scatter (SSC). The FSC provides information about cell size, while the side scatter provides information about the granularity of the cells (the internal complexity). This gives the opportunity to discriminate between cells when the information is sent to a computer. The lights from the optical system are recorded by **light detectors** and converted by an **electronic system** and can hereafter be presented on a **computer** as either single parameter histograms or as two‐parameter plots called cytograms. *Created with BioRender.*

Flow cytometry (Figure 10) is a technique used for measuring the chemical or physical characteristics of cells or particles. The cells or particles of interest are suspended in solution, labelled with fluorescent markers, and run through a cell analyser ‐ a flow cytometer instrument. The cells or particles flow through a small tube one at a time, and a laser is focused on them, scattering light and fluorescence of different wavelengths then recorded by the flow cytometer. Data are presented on a computer as either single parameter histograms or two‐parameter plots called cytograms.

The protocol describing handling and preparation of the blood samples before running on the flow cytometer is described thoroughly in papers II, III and IV (Appendix A page 35).

We used a BD FACSCantoII flow cytometer (BD Biosciences, Franklin Lakes, NJ, USA), and to analyse the data, we used the software Kaluza Analysis (Kaluza Analysis version 2.1; Beckman Coulter, Inc., Pasadena, CA, USA). For measuring white blood cell count (WBC) to obtain 1.0 x 10^6^ leukocytes in the test tubes, we used a Sysmex KX‐21NTM (Sysmex Corporation, Kobe, Japan).

Table 5 is an overview of the monoclonal antibodies used for flow cytometry. A gating strategy for the flow cytometric analyses is shown in paper IV (page 35).

**TABLE 5:** Monoclonal antibodies and corresponding isotype controls used for flow cytometric analyses

Monoclonal antibody					Negative isotype controls	
Marker	Fluorochrome	Isotype	Cat. No.	Manufacturer	Cat. No	Manufacturer
CD4	Peridinin‐Chlorophyll‐Protein	IgG2a	FAB3791C	R&D Systems	400232	BioLegend
CD8	Phycoerythrin‐Cyanine7	IgG1	300914	BioLegend	400126	BioLegend
CD8	Brilliant Violet V510	IgG1	301048	BioLegend	400172	BioLegend
CD14	Pacific Blue	IgG1	325616	BioLegend	400151	BioLegend
CD16	Brilliant Violet V510	IgG1	302048	BioLegend	400172	BioLegend
CD16	Allophycicyanine‐Cyanine7	IgG1	302018	BioLegend	400128	BioLegend
CCR7	Brilliant Violet V510	IgG2a	353232	BioLegend	400268	BioLegend
CD45Ra	Pacific Blue	IgG2b	304123	BioLegend	400331	BioLegend
CD45Ro	Fluorescein isothiocyanate	IgG2a	MCA461FT	Bio‐Rad	IC003F	R&D Systems
CD27	Phycoerythrin	IgG1	356406	BioLegend	555749	BD Biosciences
CD28	Allophycicyanine	IgG1	302912	BioLegend	400120	BioLegend
CD56	Allophycicyanine‐Cyanine7	IgG1	318332	BioLegend	400128	BioLegend
CXCR3	Phycoerythrin‐Cyanine7	IgG1	560831	BD Biosciences	400126	BioLegend
CD35	Allophycicyanine	IgG1	FAB5748A	R&D Systems	400120	BioLegend
CD59	Phycoerythrin	IgG2a	304708	BioLegend	A09141	Beckman Coulter

#### Immunoassays

We used both singleplex and multiplex immunoassays kits (Meso Scale Discovery, Rockville, Maryland, USA), to quantify levels of cytokines, anaphylatoxins and growth factors. The multiplex assay plates allow quantification of multiple analytes in the same sample, and thus less material is needed. The analyses were done at the Technical University of Denmark (DTU), Lyngby, Denmark. Before analyses, the dilution factor of each analyte was determined. The multiplex plates were prepared according to Meso Scale's instructions. Standard curves were made using standards (with known concentrations from Meso Scale) added to the plates. The standard curve was used to determine the analyte concentration. In addition, all tests were run in duplicates, and the mean concentration and the coefficient of variation (CV: the ratio of standard deviation to the mean) was calculated. The plates were read immediately after plate preparation on a QuickPlex SQ120 (Meso Scale Discovery).

#### SNP analyses

The Kennedy Centre, Denmark, did Genomic DNA extraction from stored blood samples with Chemagic Magnetic Separation Module 1 (Chemagen, Baesweiler, Germany). The extracted DNA was shipped to BioXpedia, Aarhus, Denmark, for SNP genotyping with the Fluidigm 96.96 Dynamic Array Integrated Fluidic Circuit (Fluidigm Corp, South San Francisco, California, USA). All assays were performed according to the manufacturer's protocol. The raw data were imported to the Fluidigm SNP Genotyping Analysis software v.4.5.1 and analysed with the standard settings.

#### PCR analyses

Mutation analyses for *JAK2V617F*, *CALR*, and *MPL* was performed with highly sensitive real‐time quantitative polymerase chain reaction (PCR) on an ABI Prism7900HT (Applied Biosystems, Foster City, CA, USA). This was done on fluorescence‐activated cell sorted (FACS) monocytes, lymphocytes, and granulocytes from peripheral EDTA anticoagulated blood on a FACSVantage (BD Biosciences). The DNA was extracted using a MagnaPure robot (Roche Diagnostics, Mannheim, Germany) according to the manufacturer's protocol.

### DATA ANALYSIS

#### Patient data

All patient data were recorded in Excel spreadsheets version 16.57 (Microsoft, Redmond, Washington, USA), and images were saved in software programs (Heidelberg Eye Explorer, Heidelberg, Germany) and IMAGEnet ibase version 3.25.0 (Topcon, Tokyo, Japan). Both spreadsheets and images were kept on secure servers in Region Zealand. Informed consent forms were kept in a locked cabinet in a locked room.

#### Statistical software

For statistical analyses, we used the SAS statistical software package version 9.4 (SAS Institute Inc., Cary, North Caroline, USA) for study I and RStudio version 4.1.1. (For macOS, R Studio, Boston, Massachusetts, USA) for study II.

#### Statistical analyses

The applied statistical tests used for each substudy are described in detail in the included papers (Appendix A page 35). The description below is a summary.

*Continuous normally distributed data* are shown as mean, and 95% confidence interval (95% CI) and comparisons between groups were made using parametric tests (independent samples t‐test, one‐way analysis of variance (ANOVA), two‐way ANOVA, linear regression models). We assessed if continuous data were normally distributed visually with histograms and qq plots.

*Non‐normally distributed data* are shown as median and interquartile range (IQR), and comparisons were made using non‐parametric tests (Wilcoxon's rank‐sum test, Kruskal Wallis test, robust linear regression).

*Categorical data* are presented as numbers or percentages, and comparisons between groups were made using the Chi‐squared test or Fisher's Exact test (for expected count ≤ 5). Statistical significance was in all studies defined as p<0.05.

In study I, we also evaluated inter‐observer‐agreement in image grading by calculating Cohen's kappa coefficient.

In study II we calculated a z‐score for each of the measured pro‐inflammatory markers and averaged the scores to obtain a summary score for each patient. We calculated Cohen's *d* (the ratio between the group difference and the standard deviation) to evaluate the size of the observed differences in summary scores between groups. The effect size was interpreted as small if 0.2, moderate if 0.5, and large if 0.8.

## RESULTS AND DISCUSSION

The results described and the discussion written in this section will summarise the same sections of our published papers corresponding to each study and substudy of this thesis. The focus will be on the studies' most important or interesting findings. Detailed results and discussion sections can be found in the included papers in the Appendix section (page 35). A suggestion is to read the summaries presented in this section prior to reading each corresponding paper and hereafter reading the section “conclusion and perspectives” on page 27.

### STUDY I: RETINAL CHANGES ASSOCIATED WITH AMD IN PATIENTS WITH MPNs (PAPER‐I)

#### Introduction

The register study of Bak et al., 2020, mentioned in the background section (page 13), has shown an increased AMD risk in MPNs. However, we do not have any information on the retinal status of these patients. Study‐I of this PhD project investigated the prevalence of retinal changes associated with AMD using imaging techniques (Fundus photography and OCT). We graded the images and compared the results to the estimates from three large population‐based studies (The Beaver Dam Eye Study, The Blue Mountains Eye Study, and the Rotterdam Eye Study). The primary outcomes were drusen size (largest present), pigmentary abnormalities, AMD stages (early‐, intermediate‐ and late AMD), NLR, *JAK2V617F* allele burden, neuroretinal‐ and RPE‐BM thickness (this thickness was evaluated and compared to a previous study of a healthy control group). We also evaluated area‐covered‐by drusen, drusen count and type as secondary outcomes.

#### Results and discussion

**FIGURE 11:**
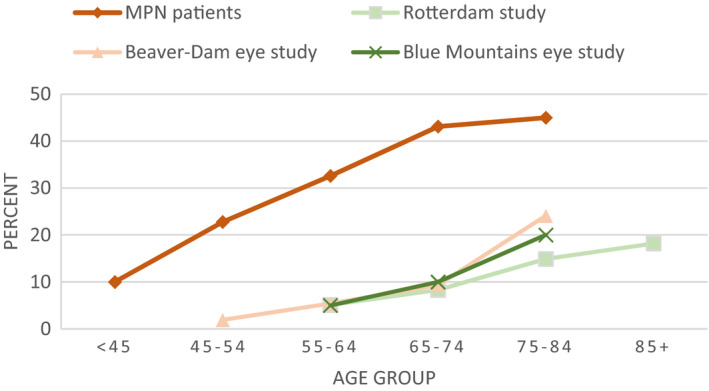
Prevalence of large drusen in patients with MPNs compared to estimates from three large population‐based studies. Comparison of the prevalence of drusen >125μm as the largest drusen present within a 3000μm radius of the fovea between patients with myeloproliferative neoplasms and three large population‐based studies (Beaver Dam Eye Study, Blue Mountains Eye Study and Rotterdam Study). Reprinted from The Lancet, EClinicalMedicine 2020; 26: 100526, Liisborg C, Nielsen MK, Hasselbalch HC, Sørensen TL, Patients with myeloproliferative neoplasms and high levels of systemic inflammation develop age‐related macular degeneration., Copyright (2020), with permission from Elsevier. https://www.thelancet.com/journals/eclinm/article/PIIS2589‐5370(20)30270‐4/fulltext#articleInformation.

Patients with MPNs have a significantly higher prevalence of large drusen than the population‐based estimates (Figure 11) and an increased prevalence of the late stages of AMD. The odds ratio for patients with MPNs having large drusen were between 5.0 (CI 4.1‐8.0) and 7.0 (CI5.0‐9.7) compared to each of the population studies. The retinal changes, in addition, appear at an earlier age. We also found a higher NLR in MPNd than MPNn (p=0.038) and thickening of the BM‐RPE complex compared to healthy controls (p=0.0014). These data support the increased risk of AMD in the register study by Bak et al. (2020). The MPNs are not only related to late AMD (neovascularization) but also to accelerated accumulation of debris (thicker BM‐RPE‐complex) and drusen presence (more drusen from an earlier age). The NLR, a marker of systemic inflammation, was found associated with drusen presence supporting a role for chronic inflammation in drusen formation and thereby the pathogenesis of AMD. Systemic inflammation will be discussed further in Study II below.

### STUDY II: IMMUNOLOGICAL SIMILARITIES BETWEEN PATIENTS WITH AMD AND MPN (PAPER‐II, ‐III AND ‐IV)

#### Introduction

Study II explored the possible immunological similarities between patients with AMD and MPNs, focusing on differences between MPN patients with and without drusen. This second study was further divided into three immunological substudies. The mentioned markers have previously been described with AMD (and some for MPN) but never coincided. Please bear in mind that all three substudies compare the four patient groups described in the Methods section: nAMD, iAMD, MPNd and MPNn.

##### Study II‐a: Inflammation (PAPER‐II)

The grade of inflammation was assessed by measuring cytokines and growth factors in serum: TNF‐α, TNF‐RII, IL‐1β, IL‐6, IL‐8, Angiopoietin 2, EGF, HGF, PDGF‐A, PDGF‐B and VEGF‐A. We combined the cytokines and growth factors into a summary inflammation score (SIS) and a summary growth factor score (SGS), respectively. With flow cytometry, we measured the expression of the complement regulatory proteins (Cregs) CD35 and CD59. In plasma, we measured the anaphylatoxins C3a and C5a. We also assessed the neutrophil‐to‐lymphocyte ratio (NLR) and the distribution of monocyte subsets. Finally, we evaluated the distribution of relevant SNPs associated with an increased risk of AMD with SNP genotyping. Both SNPs were in genes related to the complement system – the complement factor H (CFH) gene rs1061170 and the C3 gene rs2230199.

##### Study II‐b: Ageing (PAPER III)

We investigated T‐cell differentiation and ageing profile (immunosenescence). This was done with flow cytometry using the T cell markers CD4 and CD8, the differentiation markers CD45Ra, CD45Ro and CCR7, the co‐stimulatory markers CD27, CD28 and the ageing marker CD56.

##### Study II‐c: Angiogenesis (PAPER IV)

To investigate angiogenesis, we studied a receptor, CXCR3, related to the inhibition of angiogenesis. We analysed the CXCR3 expression on T cells, monocytes, and monocyte subsets with flow cytometry. With immunoassays, we measured the CXCR3 ligands, the interferon (IFN)‐γ inducible chemokines CXCL9, CXCL10 and CXCL11.

#### Results and discussion

**FIGURE 12:**
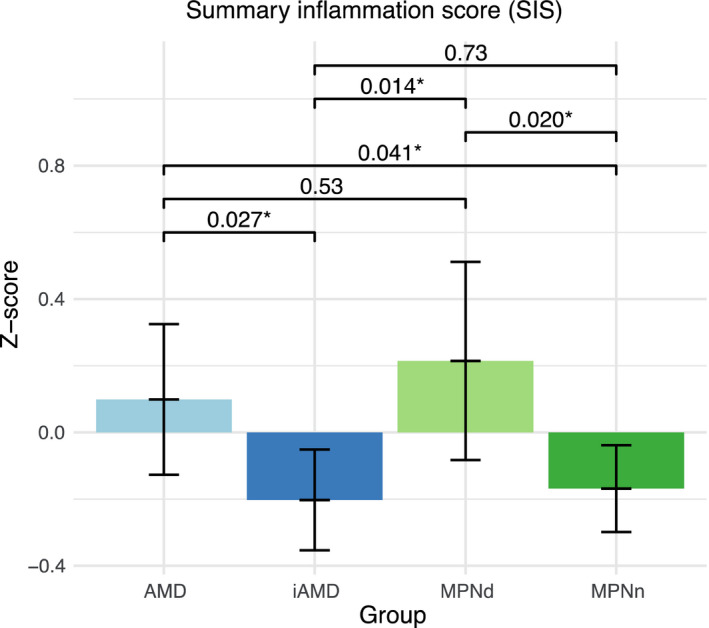
Summary inflammation score in patients with AMD and MPN Plot of summary inflammation score = ([z score (IL6) + z score (IL8) + z score (TNF‐R2) + z score (TNF‐a) + z score (IL‐1β)]) ‐ with 95% CIbars. Comparisons between patients with nAMD (n=29), iAMD (n=28), MPNd (n=35), MPNn (n=27). Reprinted from The Lancet, EClinicalMedicine 2022; 43: 201248, Liisborg C, Skov V, Kjær L, Hasselbalch HC, Sørensen TL, Patients with MPNs and retinal drusen show signs of complement system dysregulation and a high degree of chronic low‐grade inflammation, Copyright (2022), with permission from Elsevier. https://www.thelancet.com/journals/eclinm/article/PIIS2589‐5370(21)00529‐0/fulltext. iAMD, intermediate AMD; MPNd, myeloproliferative neoplasms with drusen; MPNn, myeloproliferative neoplasms with normal retinas; nAMD, neovascular AMD.

**FIGURE 13:**
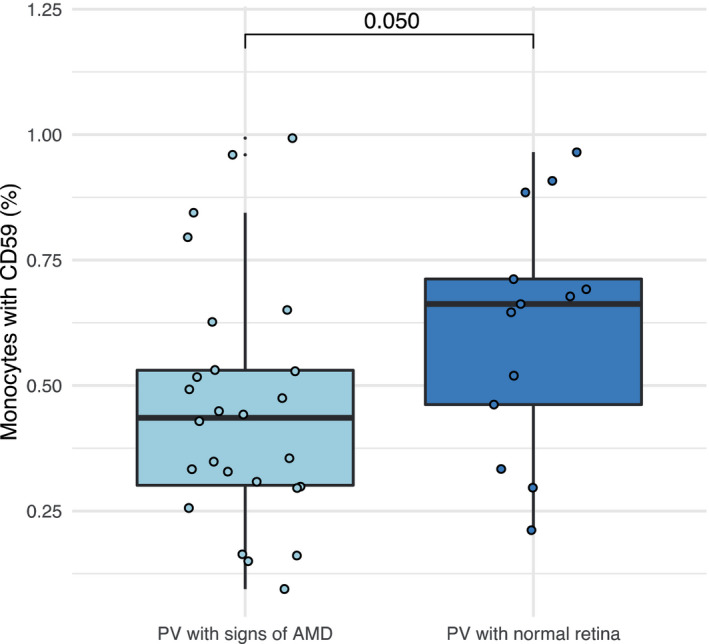
Expression of CD59 in patients with PV. Expression of complement regulatory protein CD59 in patients with polycythemia vera with (n=26) and without drusen (n=13). Reprinted from The Lancet, EClinicalMedicine 2022; 43: 201248, Liisborg C, Skov V, Kjær L, Hasselbalch HC, Sørensen TL, Patients with MPNs and retinal drusen show signs of complement system dysregulation and a high degree of chronic low‐grade inflammation, Copyright (2022), with permission from Elsevier. https://www.thelancet.com/journals/eclinm/article/PIIS2589‐5370(21)00529‐0/fulltext. PV, Polycytheamia vera.

In study II‐a, we found a higher SIS in patients with MPNd compared to MPNn (p=0.020). When we compared MPN and AMD, we found that MPNd SIS resembled the SIS in nAMD while MPNn and iAMD had lower scores and looked similar (Figure 12). We found no difference between the groups when observing single cytokines, but the differences between groups were significant when evaluating the SIS, indicating that a general rise in the cytokines in nAMD and MPNd are present. However, the differences are not significant when looking at individual cytokines. We also found evidence of a dysregulated systemic complement system in MPNs when investigating complement regulatory proteins (Cregs). Despite not reaching significance, Cregs expression in MPNn seemed much lower than MPNd, and when we subdivided MPNs into subtypes, we found that PV patients had a lower expression than ET (p=0.027) and PMF (p=0.016), and those with drusen had a significantly lower expression than those without drusen (p=0.050), (Figure 13). A previous study has found lower Cregs expression in nAMD (Singh et al., 2012). In addition, we also investigated the NLR between groups and found similar results, with MPNd, having higher NLR than MPNn and the two AMD groups. These results suggest a possible role for low‐grade systemic inflammation in AMD pathogenesis. Therefore, we hypothesise that chronic low‐grade inflammation triggers drusen formation and leads to more inflammation, creating a vicious cycle with more drusen, which increases the risk of developing AMD. A recent study of patients with iAMD have found similar results of systemic alterations supporting a role for systemic inflammation in iAMD and that combinations of inflammatory factors in plasma were associated with progression to late AMD (Wagner et al., 2021).

Several other studies support the hypothesis of systemic inflammation in AMD pathogenesis. Several studies of inflammatory diseases have reported increased prevalence or an association with AMD (Schnabolk, 2019; Schnabolk et al., 2019). For instance, patients with antiretroviral‐treated HIV have a 4‐fold increase in iAMD (and other age‐related diseases) compared to an age‐matched non‐HIV infected control group. These antiretroviral‐treated patients do not have a normal immune system, but they have immunosenescence changes (similar to an older non‐HIV control group), and a state of chronic low‐grade inflammation also characterises them. The study proposes that this systemic inflammation and/or immunosenescence may cause the increased iAMD prevalence. Further, studies have shown that inflammation is involved in many diseases previously not regarded as inflammatory disorders, such as atherosclerosis (Hansson, 2009; Hansson et al., 2006; Stoll & Bendszus, 2006), obesity and diabetes (Ellulu et al., 2017; Mathieu et al., 2010). Evidence points to systemic inflammation playing a significant role in neurodegenerative diseases or diseases that occur in “immune privileged” tissues (Alzheimer's, Parkinson's) (Adams et al., 2019; Holmes, 2013; Walker et al., 2019). In addition, as described in the inflammation section on page 13, patients with nAMD tend to have higher levels of plasma anaphylatoxins and other complement components (Lechner et al., 2016; Lynch, Mandava, et al., 2020a; Lynch, Palestine, et al., 2020b; Lynch, Wagner, et al., 2020c; Machalińska et al., 2009; Reynolds et al., 2009; Scholl et al., 2008; Sivaprasad et al., 2007), increased CRP (Hong et al., 2011; Mitta et al., 2013) and higher inflammatory cytokine levels (Liukkonen et al., 2022; Nassar et al., 2015). Also, increased levels of matrix metalloproteases (MMPs), which are involved in extracellular matrix (ECM) remodelling and lower concentration of their regulators, have been observed (Chau et al., 2007; Krogh Nielsen et al., 2019). The systemic increased levels of pro‐inflammatory cytokines may activate macrophages and their release of MMPs, influencing ECM of the BM. The other systemic factors of chronic inflammation mentioned could also disrupt the normal cell function by driving cell differentiation, causing immunosenescent changes (described further below), and activating the endothelium and thereby attracting effector cells of the immune system. Finally, but not least, many of the polymorphisms associated with increased risk of AMD are in genes playing a role in the immune system and, in particular, the complement system.

**FIGURE 14:**
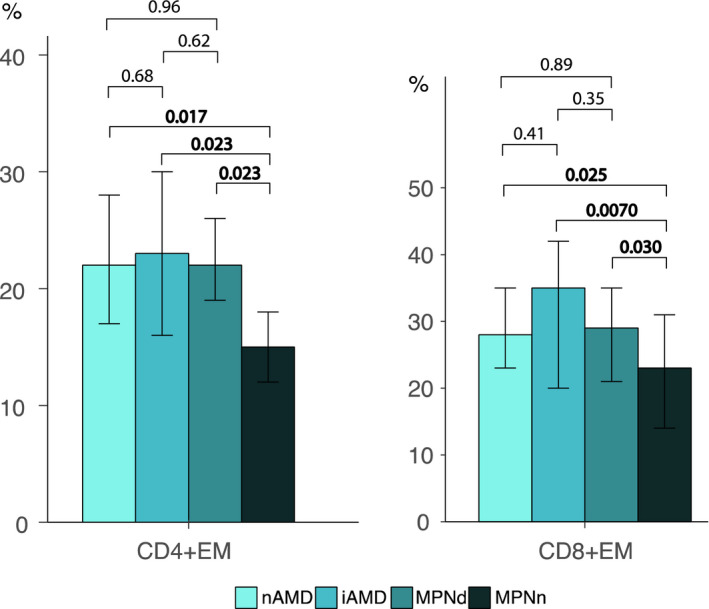
Effector memory cells in patients with AMD and MPNs. MPNn patients stand out with lower percentage of effector memory T cells. Reprinted from Impact Journals, LLC, Aging 2021; 13: 25763–25777, Liisborg C, Skov V, Kjær L, Hasselbalch HC, Sørensen TL, Retinal drusen in patients with chronic myeloproliferative blood cancers are associated with an increased proportion of senescent T cells and signs of an ageing immune system, Copyright (2021), with permission from Impact Journals, LC. https://www.aging‐us.com/full/13/25763. iAMD, intermediate AMD; MPNd, patients having myeloproliferative neoplasms with drusen; MPNn, patients having myeloproliferative neoplasms having normal retinas; nAMD, neovascular AMD.

Study II‐b found evidence of accelerated ageing changes (immunosenescence) in MPNd compared to MPNn. The data suggest that patients with MPNd have a differentiation profile resembling nAMD and iAMD and significantly more effector memory T cells than MPNn in both the CD4+ and CD8+ compartment. From Figure 14, it is evident that MPNn stands out from the other groups. Another interesting finding in this study was that more terminally differentiated T cells (EMRA T Cells) seemed to accumulate over the biological MPN continuum. Patients with myelofibrosis had a significantly higher percentage of EMRA than ET (p=0.024) and near significantly higher than PV (0.070) (Paper III, Figure 3).

Inflammation and ageing are interconnected processes. An inflammatory environment with continuous release of inflammation products and elevated ROS levels leading to DNA damage can induce senescence. Senescent T cells can be regarded as synonymous with effector T cells. Senescent cells do not proliferate, but they are very active and secrete various cytokines and cytotoxic granules, a well‐known phenomenon referred to as the senescence‐associated secretory phenotype (SASP). The cells with a SASP are highly cytotoxic cells capable of destructing healthy tissue, as well as they are thought to play a role in inflamm‐ageing (Coppé et al., 2010; Franceschi et al., 2006).

**FIGURE 15:**
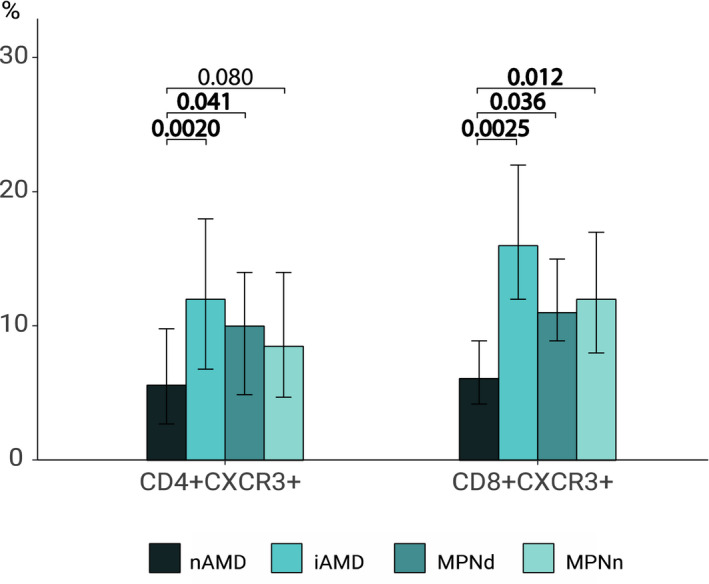
CXCR3 expression T cells in patients with AMD and MPNs. A significantly lower CXCR3 expression in CD4+ and CD8+ t cells is found in the nAMD group compared to the other groups. Reprinted from Impact Journals, LLC, Aging 2021; 13: 25763‐25777, Liisborg C, Skov V, Kjær L, Hasselbalch HC, Sørensen TL, Retinal drusen in patients with chronic myeloproliferative blood cancers are associated with an increased proportion of senescent T cells and signs of an ageing immune system, Copyright (2021), with permission from Impact Journals https://www.aging‐us.com/full/13/25763. iAMD, intermediate AMD; MPNd, patients having myeloproliferative neoplasms with drusen; MPNn, patients having myeloproliferative neoplasms having normal retinas; nAMD, neovascular AMD.

**FIGURE 16:**
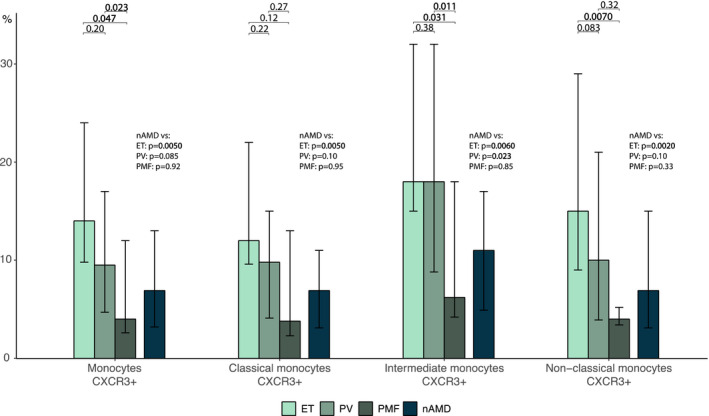
Expression of CXCR3 in subtypes of MPNs and neovascular AMD. A decreased CXCR3 expression is observed over the MPN biological continuum. Reprinted from Impact Journals, LLC, Aging 2021; 13: 25763‐25777, Liisborg C, Skov V, Kjær L, Hasselbalch HC, Sørensen TL, Retinal drusen in patients with chronic myeloproliferative blood cancers are associated with an increased proportion of senescent T cells and signs of an ageing immune system, Copyright (2021), with permission from Impact Journals, LCC. https://www.aging‐us.com/full/13/25763. ET, essential thrombocythemia; nAMD, neovascular AMD; PMF, primary myelofibrosis; PV, polycythemia vera.

Study II‐c showed a significantly lower CXCR3 expression on T cells (Figure 15) and some monocyte subsets in nAMD compared to the other three groups (Paper IV, Figure 2), supporting previous findings from our group of lower CXCR3 expression in nAMD compared to healthy controls. We did not find any difference between the MPNd and MPNn groups, but since the patients with MPNd only have early or intermediate AMD, it may not come as a surprise that we observe no difference since angiogenesis occurs in nAMD. However, the MPNd groups include both eAMD and iAMD, so it could be relevant to study CXCR3 in patients with iAMD. We also found a decreasing expression of CXCR3 in most monocyte subsets over the biological MPN continuum from ET to PV to MF (Figure 16). Very low expression was observed in the advanced myelofibrosis stage, a disease which in the bone marrow shares characteristics seen in the retina in patients with nAMD: angiogenesis and fibrosis. The expression was similar in PMF and nAMD. These results support dysregulation of the CXCR3 receptor in both AMD and PMF pathogenesis. The nAMD level was inserted in Figure 16. to compare the levels with the MPN subtypes.

**FIGURE 17:**
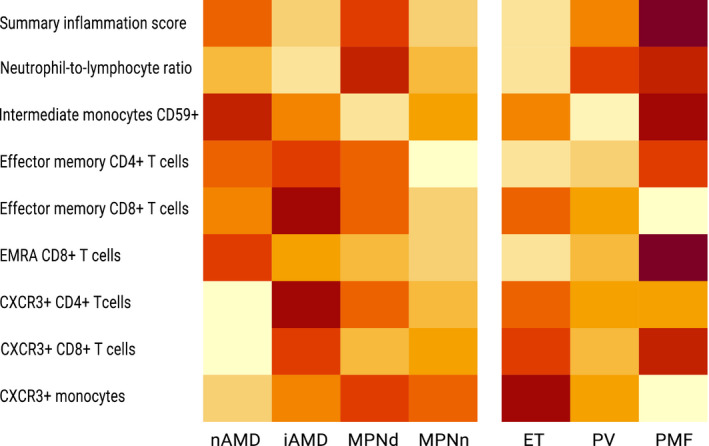
Heatmap of selected significant results from study II. Heatmap showing levels of selected markers from study II. Colour coding is done from lowest to highest measured level in the four groups for each marker. Dark colours represent high levels, while light colours represent low levels.

In summary, substudy II (a‐c) show systemic differences between patients with MPN with and without drusen (except for in study II‐c), supporting that these alterations in the systemic circulation (markers of inflammation, ageing and angiogenesis) are a part of the AMD pathogenesis. Our findings are summarised in (Figure 17).

We speculate that especially systemic inflammation and immunosenescence changes play a more critical part in the pathogenesis. The current understanding or theory on how drusen develop is the accumulation of oxidative insults that increase with age, and oxidative damage is believed to be the initial trigger of AMD. The retinal tissue is highly metabolically active with large oxygen consumption, exposed to a large amount of light, and have a high content of polyunsaturated fatty acids, resulting in a high sensitivity to oxidative stress (Kaarniranta et al., 2013; Zhang et al., 2020). In addition, the RPE and neuroretina are non‐replicative, terminally differentiated cells. The accumulating damage exhausts the local autonomous cell response, with decreased autophagy, and the stressed cells may undergo senescence or die. Senescent cells may secrete inflammatory cytokines and chemokines (maybe another example of SASP), activating the local immune regulating system. If the damage/stress again exceeds the capacity, this will eventually lead to decreased phagocytosis resulting in loss of clearance and lipid accumulation. The microglia and macrophages of the local regulatory system may release additional cytokines and chemokines, which reach the systemic circulation and activate the systemic immune system. The earliest pathological changes are the appearance of basal deposits called basal laminar deposits (BLamD) and basal linear deposits (BLinD) and thickening of BM with reduced permeability (Ambati et al., 2003; Curcio et al., 2011). Hereafter, drusen develop. The systemic inflammatory response is thought to be secondary to BM destruction (Chen & Xu, 2015).

**FIGURE 18:**
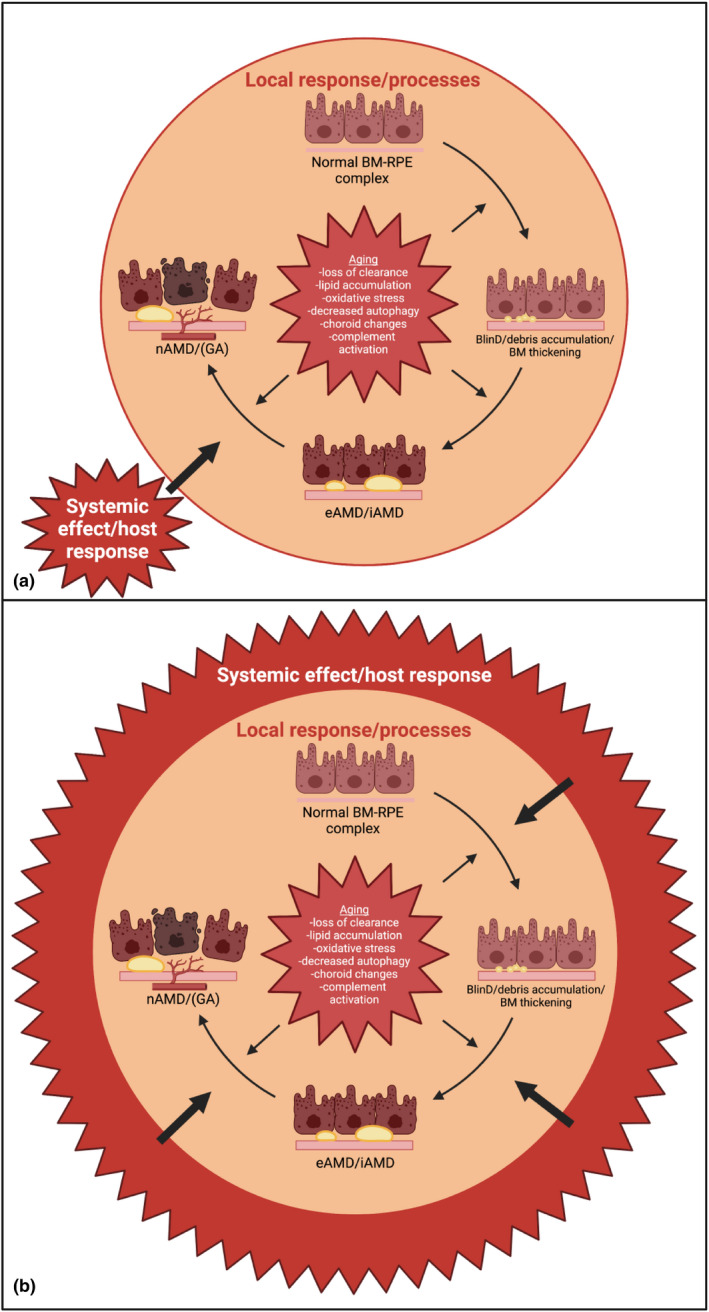
AMD models The current understanding of how drusen develop are the accumulation of oxidative insults and debris that increase with age. Oxidative damage is believed to be the initial trigger of AMD. The highly metabolically active retinal tissue are exposed to a large amount of light and have a high content of polyunsaturated fatty acids, resulting in a high sensitivity to oxidative stress. The accumulating damage exhausts the local autonomous cell response, with decreased autophagy, and the stressed cells may undergo senescence or die. Senescent cells may secrete inflammatory cytokines and chemokines activating the local immune regulating system, and if the damage/ stress again exceeds the capacity, this will eventually lead to decreased phagocytosis resulting loss of clearance and lipid accumulation. The microglia and macrophages of the local regulatory system may release additional cytokines and chemokines which reach the systemic circulation and activate the systemic immune system. The earliest pathological changes are the appearance of basal deposits called basal laminar deposits (BLamD) and basal linear deposits (BLinD) and thickening of BM, with reduced permeability. (a) Our group have previously proposed a model for AMD development ‐ a two‐level model hypothesis”. The accumulation of age‐related ocular damage comprise the first step in the development and the subsequent inflammatory host response the second step. Depending on the host response, the progress rate varies. Adding to the complexity, a “collection” of risk factors (genetic, environmental and health behaviours) is involved in the pathogenesis and can either halt or speed up the process (not shown). (b) Based on the findings in this PhD project, we now propose that the systemic inflammation can trigger and drive the accumulation of debris that leads to drusen formation. All steps in the accumulation process can be affected by and initiated by systemic inflammation and accelerate the ageing process and the following decline in different functions and mechanisms. The systemic inflammation is capable of damaging retinal tissue and triggering the local response.

Our group have previously proposed a model for AMD development ‐ a two‐level model hypothesis” (Rozing et al., 2020). The accumulation of age‐related ocular damage comprise the first step in the development, and the subsequent inflammatory host response the second step (Figure 18a). Depending on the host response, the progress rate varies. Adding to the complexity is the involvement of a group of risk factors in the pathogenesis (genetic, environmental and health behaviours), which can either halt or speed up the process. Based on the results of this PhD project, we now propose that systemic inflammation can trigger and drive the accumulation of debris that leads to drusen formation. (Figure 18b). All steps in the accumulation process can be affected by and initiated by systemic inflammation and accelerate the ageing process and the following decline in different functions and mechanisms. Systemic inflammation is capable of damaging retinal tissue and triggering the local response. The systemic inflammation may even damage the choriocapillaris. Both histopathological studies and in vivo studies using OCT angiography could support this theory since they suggest that choriocapillaris loss precedes the neurodegeneration seen in AMD (Fleckenstein et al., 2021).

The reason some people develop AMD could be due to their systemic immune response, genetic predisposition, lifestyle, and the number of insults (inflammatory episodes, infections and other stressors or “protectors” such as anti‐inflammatory treatments) accumulating during their life. The patients with MPNs are exposed to massive systemic inflammation early in life, and therefore these processes are accelerated (including accelerated ageing).

The reason some of the patients develop neovascular AMD could also depend on systemic alterations, as the decreased leukocyte CXCR3 expression shown in study II‐c could be an example of. The retina is an immune‐privileged tissue. However, animal studies have shown that circulating CCR2+ monocytes are implicated in angiogenesis in laser‐induced CNV (Espinosa‐Heidmann et al., 2003; Sakurai et al., 2003; Sennlaub et al., 2013; Tsutsumi et al., 2003). Further, several studies have shown blood‐derived mononuclear phagocytes accumulation in the subretinal space both in animal models and in post‐mortem eyes of patients with AMD (Guillonneau et al., 2017; Gupta et al., 2003; Lad et al., 2015). Interestingly, CD8 positive T cells are also shown to be more abundant in the macular choroid of patients having drusen, providing further evidence of an important systemic involvement (Ezzat et al., 2008; Penfold et al., 1984, 1986).

Similar evidence suggesting the involvement of circulating cells in the brain, another immune‐privileged tissue, has also been shown. For instance, bone marrow chimeric studies have shown that circulating monocytes regularly replace brain perivascular macrophages (Soulas et al., 2009).

We found evidence of more effector memory T cells in patients with MPNs and drusen compared to those with normal retina. These more cytotoxic cells, the inflammatory environment and macrophages could potentially harm the choroid, BM, photoreceptors and RPE.

Limitations: Caution should be taken when interpreting the data of this PhD project. Although the drusen seen in the MPNs are clinically and photographically identical to those observed in AMD, we do not have histological data to confirm this. It may seem plausible that the drusen are similar since the patients with MPN tend to develop late AMD more often than the general population. Patients with MPNs were enrolled from the outpatient program at the haematology department and asked to participate. It is possible that patients with visual symptoms were more likely to enrol, resulting in selection bias, but also those with a heavy comorbidity burden (and more severe MPN) could be less likely to say yes to participation. It is a limitation that we do not have a healthy control group to compare the groups with, but this was intentional to avoid having too many groups and, therefore, too many multiple comparisons. In addition, the markers investigated in this project have previously been studied in nAMD and compared to healthy controls. Another aspect of including healthy controls is the great variety of the ageing process in individuals. Another limitation of the studies is the small groups of patients with ET and PMF (17 and six, respectively), but still, we found significant changes across the MPN continuum.

Due to the observational nature of the studies, we cannot infer causality. We do not know which changes/alterations came first, and further, we are investigating systemic markers in a huge and complex inter‐related system with unlimited interactions and potential confounders.

## CONCLUSION AND PERSPECTIVES

### CONCLUSIONS

The objectives and hypotheses of this PhD project were:

1. To investigate if patients with MPNs show a higher prevalence of retinal changes compatible with AMD and from an earlier age. Our null hypothesis was that patients with MPN do not differ in prevalence of drusen and AMD compared to population estimates.
  - Study I showed that patients with MPNs have a higher prevalence of drusen than the general population and from an earlier age. Consequently, they also have a higher prevalence of AMD, and the prevalence of late‐stage AMD was significantly higher in these patients. We reject the null hypothesis.
2. To assess the immunological similarities in patients with MPNs and AMD ‐ to identify previously relevant changes in the immune system found in AMD, which are shared with MPNs. More importantly, we wanted to investigate if patients with MPN and drusen show overlap with patients with AMD in these immunological signatures and are different from patients with MPNs and normal retinas. Our null hypothesis was that MPNd and MPNn do not differ in the systemic markers investigated.
  - The three substudies of study II showed that patients with MPNd are immunologically different from patients with MPNn. We observed an overlap between nAMD/(iAMD) and MPNd in immunological markers that differed between the MPNs with and without drusen, supporting that these specific changes could be a part of drusen/AMD pathogenesis. We reject the null hypothesis for the markers in studies II‐a and II‐b, but not in study II‐c. The immunological markers in question are:
    - Inflammation: we found a higher level of low‐grade systemic inflammation in MPNd than MPNn (indicated by a higher summary inflammation score and neutrophil‐to‐lymphocyte ratio, as well as signs of inadequate regulation of the complement system with lower monocyte CD59 expression in patients with PV having drusen compared to patients with PV and normal retinas.
    - Ageing: we found signs of an accelerated ageing immune system in MPNd compared to MPNn (more effector memory‐/senescent T cells).
    - Angiogenesis: we found a lower CXCR3 expression on T cells and most monocyte subsets in nAMD compared to iAMD, MPNd and MPNn. Further, we found a decreasing CXCR3 expression over the biological continuum with the lowest levels in the late myelofibrotic stage with resembling levels seen in nAMD. The disease stages (PMF and nAMD) are characterised by angiogenesis and fibrosis, supporting the role of CXCR3 in both diseases.

In summary, the findings suggest that patients with MPNs have an increased prevalence of drusen and AMD from an earlier age, likely due to their inflammatory state. The results also suggest that systemic alterations play a more critical role in AMD pathogenesis. We propose an AMD model (Figure 18) where inflammation plays an essential part in AMD pathogenesis. A model where inflammation can initiate and accelerate the normal age‐dependent accumulation of debris in the retina, leading to basal deposits, which develop into drusen and eventually lead to the changes seen with early and intermediate AMD. Finally, this results in the increased risk of developing late‐stage AMD.

### PERSPECTIVES

In developed countries, neovascular AMD is the leading cause of irreversible vision loss in the elderly population. Treatment with VEGF inhibitors has improved the outcome of many patients with a decrease in severe vision impairment and blindness (Bloch et al., 2012). Despite the benefits of anti‐VEGF therapy for nAMD, long‐term follow‐up has revealed progressive vision loss with time, owing to macular atrophy and fibrosis (Ricci et al., 2020). Also, the benefits come at a high cost, with many recurring visits to the doctor for injections, and a considerable economic burden to the health care systems with increases in drug expenditures (Day et al., 2011). The need for better treatment is evident. We also lack effective treatment for GA and especially treatments for the earlier stages and progression prevention from intermediate to late‐stage AMD. In recent years, much focus has been on modulating the alternative pathway of the complement cascade, but until now, only modest or no effect has been observed. A possible explanation could be the disease stages treated (late AMD) or insufficient drug delivery (Park et al., 2019).

AMD pathogenesis has not been fully elucidated despite intensive research, probably due to its multifactorial nature.

Although we cannot infer causality from this project's observational studies, the results described in this thesis suggest that systemic alterations play a far more critical part in AMD pathogenesis than previously anticipated. Chronic inflammation may even trigger or accelerate the age‐related debris accumulation in the body (including in the eye tissues, despite the protection from the BRB). This could open for new possibilities of treating patients with anti‐inflammatory agents and could also be the reason previous trials of, for instance, therapies targeting the complement cascade in late AMD have not shown promising results. Advanced stage AMD may be too late in the disease process to demonstrate any effectiveness, and the role of the systemic inflammatory influence keeps stimulating the local immune response in the retina. Treatments may be more effective if administered earlier at the intermediate AMD stage to forestall progression. However, one must bear in mind that these patients often do not have any symptoms and only a smaller fraction of patients with iAMD progress to late AMD (Chakravarthy et al., 2020). Therefore, there may be some ethical considerations regarding the treatment of these individuals, and further, it may be more difficult to identify individuals with iAMD since we do not see them as often in the clinic as the late stage, or at least often first when vision ‐loss or ‐disturbances are prevalent.

Few studies have investigated the significance of systemic inflammation in nAMD as described in this thesis (Lechner et al., 2016; Lynch, Mandava, et al., 2020a; Lynch, Palestine, et al., 2020b; Lynch, Wagner, et al., 2020c; Machalińska et al., 2009; Reynolds et al., 2009; Ristau et al., 2014; Scholl et al., 2008; Sivaprasad et al., 2007; Smailhodzic et al., 2012), and even fewer have addressed this in iAMD (Lynch, Mandava, et al., 2020a; Lynch, Palestine, et al., 2020b; Lynch, Wagner, et al., 2020c; Wagner et al., 2021). A recent study from 2021 has interestingly found an association between systemic inflammatory factors and progression from iAMD to late AMD (Wagner et al., 2021). However, studies on anti‐inflammatory treatment with aspirin and NSAIDs in patients with AMD have shown conflicting results, with some studies suggesting a possible exacerbating role of aspirin in nAMD, others find no effect, while again others find a modest effect of long‐term, low‐dose aspirin, reducing the risk of AMD (Nowak, 2014). Two newer studies from 2018 (Modjtahedi et al., 2018) and 2021 (Xu et al., 2021) have shown a decrease in intermediate and late‐stage AMD risk with the regular use of low dose aspirin or other NSAIDs. Similar studies and findings on statins, the cholesterol‐lowering medications which also have anti‐inflammatory properties, have shown inconclusive results (Roizenblatt et al., 2018). Again, maybe more studies explicitly designed for treating iAMD patients with anti‐inflammatory therapy are needed. It would be intriguing to treat iAMD patients with anti‐inflammatory agents with a control group receiving placebo and compare how many individuals from the two groups progress to advanced AMD, or if some agents could even initiate drusen regression. We also have the possibility of following patients with MPNs who already receive different therapies with anti‐inflammatory and immunomodulating properties (acetylsalicylic acid, statins, Hydroxyurea, interferon‐α, Ruxolitinib), comparing the prevalence and progression of AMD.

For Alzheimer's disease, observational studies have shown that inflammation occurring decades before the typical age of dementia may promote neurodegenerative and cognitive decline, but as with our studies, they cannot assess causality. However, it is found that circulating inflammatory proteins can communicate with the CNS in both neural and humoral ways, and this includes afferent Vagus nerve signalling (Walker et al., 2019). Interestingly, our group have also investigated Vagus nerve signalling, which indicated an increased risk of AMD in vagotomised patients and a possible impaired vagal tone in patients with nAMD compared to healthy controls (unpublished results). In summary, systemic inflammation is believed to increase local CNS inflammatory signalling, resulting in activation of microglia and astrocytes, quite similar to the proposed mechanisms in late AMD (Cunningham & Hennessy, 2015).

More studies of the role of systemic inflammation are needed, especially in iAMD. Understanding the role of systemic inflammation could have a tremendous clinical impact and the possibility of identifying specific markers linked to progression and high‐risk individuals. These studies could result in targeted treatments in early disease and thereby preventing progression to the late debilitating stages of AMD.

Suppose systemic inflammation plays a more crucial role. In that case, the general advice of quitting smoking, more regular physical activity and eating a healthy diet (Mediterranean) are still very favourable and important, since such lifestyle choices are associated with a lowering of systemic inflammation. Interestingly, recent meta‐analyses show the association between these lifestyle choices and a lower occurrence of AMD (Chapman et al., 2019; McGuinness et al., 2018). An exciting study in our department is in progress randomising patients with GA to physical activity and a control group to investigate if physical exercise can halt the progression of GA.

Other attractive future projects come to mind.

- The MPNs can be used to study or find other relevant markers of AMD. In study I, we found more patients with GA than nAMD. Could this be because of the patients immunomodulating treatment preventing them from turning into nAMD, or do patients with MPNs have systemic changes simulating the systemic environment in patients with GA? Studies comparing markers associated with GA with MPNs would be interesting.
- For the MPNs, mathematical modelling has been done and adds further proof to the concept of chronic inflammation as a trigger and driver of these diseases (Andersen et al., 2017). Investigating if similar modelling could be done regarding systemic inflammation and drusen presence/development would be intriguing.
- Exploring neutrophil involvement and function in MPNs with and without drusen (and compared to AMD) could also be a possibility since neutrophil involvement in AMD pathogenesis has received increased attention (Ghosh et al., 2019; Martínez‐Alberquilla et al., 2022). Neutrophils change with ageing, and in vitro studies have demonstrated abnormal trafficking, lower phagocytic activity, and a higher production of intracellular ROS. Further, some studies also suggest altered neutrophil expression due to systemic inflammation, which also could affect their function (Alonso‐Fernández et al., 2008; Drew et al., 2018).
- Regarding our results of decreased CXCR3 expression, more basic studies investigating the role of CXCR3 in the retina would be exciting and clinical studies investigating CXCR3 in patients with intermediate AMD.

Finally, our studies have some clinical implications. It is important to be observant of ophthalmological symptoms in patients with MPNs (and maybe in general other inflammatory diseases). This observance also applies the other way around. If patients show drusen or AMD early in life, it could be relevant to examine if an underlying systemic or inflammatory disease could be the cause.

## Academic supervisors and assessment committée

**Graduate programme:** Immunology and infectious diseases, Graduate School of Health and Medical Sciences, University of Copenhagen, Denmark

**Principal supervisor:** Torben Lykke Sørensen, MD, PhD, Professor, Department of Ophthalmology, Zealand University Hospital – Roskilde, Denmark and Department of Clinical Medicine, University of Copenhagen, Denmark

**Co‐supervisor:** Hans Carl Hasselbalch, MD, PhD, Professor, Department of Haematology, Zealand University Hospital – Roskilde, Denmark and Department of Clinical Medicine, University of Copenhagen, Denmark

**Assessment committee:**

**Chairman**: Christian Thomas Brandt, MD, Clinical Research Associated Professor, Department of Medicine, Zealand University Hospital – Roskilde, Denmark and Department of Clinical Medicine, University of Copenhagen, Denmark

**External assessor from Denmark:** Jesper Stentoft, MD, PhD, Professor, Department of Clinical Medicine, Aarhus University Hospital, Denmark and Department of Clinical Medicine, Aarhus University, Denmark

**External assessor from abroad:** Kai Kaarniranta, Chief Physician, MD, PhD, Professor, University of Eastern Finland and Department of Ophthalmology, Kuopio University Hospital, Kuopio, Finland

## APPENDIX A: PAPERS INCLUDED IN THE THESIS

All included papers are open access

### PAPER I

Liisborg C, Nielsen MK, Hasselbalch HC, Sørensen TL. Patients with myeloproliferative neoplasms and high levels of systemic inflammation develop age‐related macular degeneration. *EClinicalMedicine*. 2020;26:100526. doi:10.1016/j.eclinm.2020.100526

https://www.thelancet.com/journals/eclinm/article/PIIS2589‐5370(20)30270‐4/fulltext#seccesectitle0011

### PAPER II

Liisborg C, Skov V, Kjær L, Hasselbalch HC, Sørensen TL. Patients with MPNs and retinal drusen show signs of complement system dysregulation and a high degree of chronic low‐grade inflammation. *eClinicalMedicine*. 2022;43:101248. doi:10.1016/j.eclinm.2021.101248

https://www.thelancet.com/journals/eclinm/article/PIIS2589‐5370(21)00529‐0/fulltext

### PAPER III

Liisborg C, Skov V, Kjær L, Hasselbalch HC, Sørensen TL. Retinal drusen in patients with chronic myeloproliferative blood cancers are associated with an increased proportion of senescent T cells and signs of an aging immune system. *Aging (Albany NY)*. 2021;13(24):25763‐25777. doi:10.18632/aging.203803

https://www.aging‐us.com/article/203803/text

### PAPER IV

Liisborg C, Skov V, Kjær L, Hasselbalch HC, Lykke Sørensen T. Lower CXCR3 expression in both patients with neovascular AMD and advanced stages of chronic myeloproliferative blood cancers. PLoS One 2022;17:e0269960. https://doi.org/10.1371/journal.pone.0269960.

https://journals.plos.org/plosone/article?id=10.1371/journal.pone.0269960

## References

[aos15247-bib-0001] Adams, B. , Nunes, J.M. , Page, M.J. , Roberts, T. , Carr, J. , Nell, T.A. et al. (2019) Parkinson's disease: a systemic inflammatory disease accompanied by bacterial inflammagens. Frontiers in Aging Neuroscience, 11, 210. 31507404 10.3389/fnagi.2019.00210 PMC6718721

[aos15247-bib-0002] Alonso‐Fernández, P. , Puerto, M. , Maté, I. , Ribera, J.M. & De La Fuente, M. (2008) Neutrophils of centenarians show function levels similar to those of young adults. Journal of the American Geriatrics Society, 56, 2244–2251. 19093924 10.1111/j.1532-5415.2008.02018.x

[aos15247-bib-0003] Ambati, J. , Ambati, B.K. , Yoo, S.H. , Ianchulev, S. & Adamis, A.P. (2003) Age‐related macular degeneration: etiology, pathogenesis, and therapeutic strategies. Survey of Ophthalmology, 48, 257–293. 12745003 10.1016/s0039-6257(03)00030-4

[aos15247-bib-0004] Ambati, J. , Atkinson, J.P. & Gelfand, B.D. (2013) Immunology of age‐related macular degeneration. Nature Reviews. Immunology, 13, 438–451. 10.1038/nri3459 PMC3941009 23702979

[aos15247-bib-0005] Andersen, M. , Sajid, Z. , Pedersen, R.K. , Gudmand‐Hoeyer, J. , Ellervik, C. , Skov, V. et al. (2017) Mathematical modelling as a proof of concept for MPNs as a human inflammation model for cancer development. PLoS One, 12, 1–18. 10.1371/journal.pone.0183620 PMC5578482 28859112

[aos15247-bib-0006] Anderson, D.H. , Mullins, R.F. , Hageman, G.S. & Johnson, L.V. (2002) A role for local inflammation in the formation of drusen in the aging eye. American Journal of Ophthalmology, 134, 411–431. 12208254 10.1016/s0002-9394(02)01624-0

[aos15247-bib-0007] Anderson, D.H. , Radeke, M.J. , Gallo, N.B. , Chapin, E.A. , Johnson, P.T. , Curletti, C.R. et al. (2010) The pivotal role of the complement system in aging and age‐related macular degeneration: hypothesis re‐visited. Progress in Retinal and Eye Research, 29, 95–112. 19961953 10.1016/j.preteyeres.2009.11.003 PMC3641842

[aos15247-bib-0008] Bak, M. , Jess, T. , Flachs, E.M. , Zwisler, A.‐D. , Juel, K. & Frederiksen, H. (2020) Risk of inflammatory bowel disease in patients with chronic myeloproliferative neoplasms: a Danish Nationwide Cohort Study. Cancers, 12, 2700. 32967227 10.3390/cancers12092700 PMC7564361

[aos15247-bib-0009] Bak, M. , Sørensen, T.L. , Flachs, E.M. , Zwisler, A.‐D. , Juel, K. , Frederiksen, H. et al. (2017) Age‐related macular degeneration in patients with chronic myeloproliferative neoplasms. JAMA Ophthalmology, 135, 835–843. 28655032 10.1001/jamaophthalmol.2017.2011 PMC5710292

[aos15247-bib-0010] Balestrieri, M.L. , Balestrieri, A. , Mancini, F.P. & Napoli, C. (2008) Understanding the immunoangiostatic CXC chemokine network. Cardiovascular Research, 78, 250–256. 18252760 10.1093/cvr/cvn029

[aos15247-bib-0011] Balkwill, F. & Mantovani, A. (2001) Inflammation and cancer: back to Virchow? Lancet, 357, 539–545. 11229684 10.1016/S0140-6736(00)04046-0

[aos15247-bib-0012] Barbui, T. , Thiele, J. , Gisslinger, H. , Kvasnicka, H.M. , Vannucchi, A.M. , Guglielmelli, P. et al. (2018) The 2016 WHO classification and diagnostic criteria for myeloproliferative neoplasms: document summary and in‐depth discussion. Blood Cancer Journal, 8, 15. 29426921 10.1038/s41408-018-0054-y PMC5807384

[aos15247-bib-0013] Barcellini, W. , Iurlo, A. , Radice, T. , Imperiali, F.G. , Zaninoni, A. , Fattizzo, B. et al. (2013) Increased prevalence of autoimmune phenomena in myelofibrosis: relationship with clinical and morphological characteristics, and with immunoregulatory cytokine patterns. Leukemia Research, 37, 1509–1515. 24080022 10.1016/j.leukres.2013.09.001

[aos15247-bib-0014] Barosi, G. (2014) An immune dysregulation in MPN. Current Hematologic Malignancy Reports, 9, 331–339. 25139710 10.1007/s11899-014-0227-0

[aos15247-bib-0015] Barosi, G. , Rosti, V. , Bonetti, E. , Campanelli, R. , Carolei, A. , Catarsi, P. et al. (2012) Evidence that prefibrotic myelofibrosis is aligned along a clinical and biological continuum featuring primary myelofibrosis. PLoS One, 7, e35631. 22536419 10.1371/journal.pone.0035631 PMC3334973

[aos15247-bib-0016] Bektas, A. , Schurman, S.H. , Sen, R. & Ferrucci, L. (2017) Human T cell immunosenescence and inflammation in aging. Journal of Leukocyte Biology, 102, 977–988. 28733462 10.1189/jlb.3RI0716-335R PMC5597513

[aos15247-bib-0017] Blann, A.D. (2000) Endothelial cell activation, injury, damage and dysfunction: separate entities or mutual terms? Blood Coagulation & Fibrinolysis, 11, 623–630. 11085282 10.1097/00001721-200010000-00006

[aos15247-bib-0018] Bloch, S.B. , Larsen, M. & Munch, I.C. (2012) Incidence of legal blindness from age‐related macular degeneration in denmark: year 2000 to 2010. American Journal of Ophthalmology, 153, 209–213. 22264944 10.1016/j.ajo.2011.10.016

[aos15247-bib-0019] Bourne, R.R.A. , Jonas, J.B. , Bron, A.M. , Cicinelli, M.V. , Das, A. , Flaxman, S.R. et al. (2018) Prevalence and causes of vision loss in high‐income countries and in Eastern and Central Europe in 2015: magnitude, temporal trends and projections. The British Journal of Ophthalmology, 102, 575–585. 29545417 10.1136/bjophthalmol-2017-311258 PMC5909755

[aos15247-bib-0020] Calle, M.C. & Fernandez, M.L. (2012) Inflammation and type 2 diabetes. Diabetes & Metabolism, 38, 183–191. 22252015 10.1016/j.diabet.2011.11.006

[aos15247-bib-0021] Callender, L.A. , Carroll, E.C. , Beal, R.W.J. , Chambers, E.S. , Nourshargh, S. , Akbar, A.N. et al. (2018) Human CD8+ EMRA T cells display a senescence‐associated secretory phenotype regulated by p38 MAPK. Aging Cell, 17, e12675 (page1‐9). 29024417 10.1111/acel.12675 PMC5770853

[aos15247-bib-0022] Campos, C. , Pera, A. , Sanchez‐Correa, B. , Alonso, C. , Lopez‐Fernandez, I. , Morgado, S. et al. (2014) Effect of age and CMV on NK cell subpopulations. Experimental Gerontology, 54, 130–137. 24440462 10.1016/j.exger.2014.01.008

[aos15247-bib-0023] Cavalloni, C. , Rumi, E. , Ferretti, V.V. , Pietra, D. , Roncoroni, E. , Bellini, M. et al. (2017) Sequential evaluation of CALR mutant burden in patients with myeloproliferative neoplasms. Oncotarget, 8, 33416. 28422716 10.18632/oncotarget.16797 PMC5464878

[aos15247-bib-0024] Chakravarthy, U. , Bailey, C.C. , Scanlon, P.H. , McKibbin, M. , Khan, R.S. , Mahmood, S. et al. (2020) Progression from early/intermediate to advanced forms of age‐related macular degeneration in a large UK cohort: rates and risk factors. Ophthalmology Retina, 4, 662–672. 32144084 10.1016/j.oret.2020.01.012

[aos15247-bib-0025] Chakravarthy, U. , Wong, T.Y. , Fletcher, A. , Piault, E. , Evans, C. , Zlateva, G. et al. (2010) Clinical risk factors for age‐related macular degeneration: a systematic review and meta‐analysis. BMC Ophthalmology, 10, 31. 21144031 10.1186/1471-2415-10-31 PMC3009619

[aos15247-bib-0026] Chan, W.K. , Rujkijyanont, P. , Neale, G. , Yang, J. , Bari, R. , Das Gupta, N. et al. (2013) Multiplex and genome‐wide analyses reveal distinctive properties of KIR+ and CD56+ T cells in human blood. Journal of Immunology, 191, 1625–1636. 10.4049/jimmunol.1300111 PMC4275795 23858032

[aos15247-bib-0027] Chapman, N.A. , Jacobs, R.J. & Braakhuis, A.J. (2019) Role of diet and food intake in age‐related macular degeneration: a systematic review. Clinical & Experimental Ophthalmology, 47, 106–127. 29927057 10.1111/ceo.13343

[aos15247-bib-0028] Chau, K.Y. , Sivaprasad, S. , Patel, N. , Donaldson, T.A. , Luthert, P.J. & Chong, N.V. (2007) Plasma levels of matrix metalloproteinase‐2 and ‐9 (MMP‐2 and MMP‐9) in age‐related macular degeneration. Eye, 2112(21), 1511–1515. 10.1038/sj.eye.6702722 17304258

[aos15247-bib-0029] Chen, M. , Lechner, J. , Zhao, J. , Toth, L. , Hogg, R. , Silvestri, G. et al. (2016) STAT3 activation in circulating monocytes contributes to neovascular age‐related macular degeneration. Current Molecular Medicine, 16, 412–423. 27009107 10.2174/1566524016666160324130031 PMC4839497

[aos15247-bib-0030] Chen, M. , Luo, C. , Zhao, J. , Devarajan, G. & Xu, H. (2018) Immune regulation in the aging retina. Progress in Retinal and Eye Research, 69, 159–172. 30352305 10.1016/j.preteyeres.2018.10.003 PMC6373845

[aos15247-bib-0031] Chen, M. & Xu, H. (2015) Parainflammation, chronic inflammation, and age‐related macular degeneration. Journal of Leukocyte Biology, 98, 713–725. 26292978 10.1189/jlb.3RI0615-239R PMC4733662

[aos15247-bib-0032] Chew, E.Y. , Clemons, T.E. , SanGiovanni, J.P. , Launer, L.J. , Grodstein, F. , Bernstein, P.S. et al. (2013) Lutein + zeaxanthin and omega‐3 fatty acids for age‐related macular degeneration: the Age‐Related Eye Disease Study 2 (AREDS2) randomized clinical trial. JAMA, 309, 2005–2015. 23644932 10.1001/jama.2013.4997

[aos15247-bib-0033] Christensen, A.S. , Møller, J.B. & Hasselbalch, H.C. (2014) Chronic kidney disease in patients with the Philadelphia‐negative chronic myeloproliferative neoplasms. Leukemia Research, 38, 490–495. 24630365 10.1016/j.leukres.2014.01.014

[aos15247-bib-0034] Christensen, K. , McGue, M. , Petersen, I. , Jeune, B. & Vaupel, J.W. (2008) Exceptional longevity does not result in excessive levels of disability. Proceedings of the National Academy of Sciences of the United States of America, 105, 13274–13279. 18711139 10.1073/pnas.0804931105 PMC2517602

[aos15247-bib-0035] Colotta, F. , Allavena, P. , Sica, A. , Garlanda, C. & Mantovani, A. (2009) Cancer‐related inflammation, the seventh hallmark of cancer: links to genetic instability. Carcinogenesis, 30, 1073–1081. 19468060 10.1093/carcin/bgp127

[aos15247-bib-0036] Coppé, J.‐P. , Desprez, P.‐Y. , Krtolica, A. & Campisi, J. (2010) The senescence‐associated secretory phenotype: the dark side of tumor suppression. Annual Review of Pathology, 5, 99–118. 10.1146/annurev-pathol-121808-102144 PMC4166495 20078217

[aos15247-bib-0037] Cordua, S. , Kjaer, L. , Skov, V. , Pallisgaard, N. , Hasselbalch, H.C. & Ellervik, C. (2019) Prevalence and phenotypes of JAK2 V617F and calreticulin mutations in a Danish general population. Blood, 134, 469–479. 31217187 10.1182/blood.2019001113

[aos15247-bib-0038] Coussens, L.M. & Werb, Z. (2002) Inflammation and cancer. Nature, 420, 860–867. 12490959 10.1038/nature01322 PMC2803035

[aos15247-bib-0039] Cunningham, C. & Hennessy, E. (2015) Co‐morbidity and systemic inflammation as drivers of cognitive decline: new experimental models adopting a broader paradigm in dementia research. Alzheimer's Research & Therapy, 7, 33. 10.1186/s13195-015-0117-2 PMC4369837 25802557

[aos15247-bib-0040] Curcio, C.A. , Johnson, M. , Rudolf, M. & Huang, J.D. (2011) The oil spill in ageing Bruch membrane. British Journal of Ophthalmology, 95, 1638. 21890786 10.1136/bjophthalmol-2011-300344 PMC3633599

[aos15247-bib-0041] Danish National Chronic Myeloid Neoplasia Study Group ; 2015. *Annual Chronic Myeloid Neoplasia Report*. *Danish* .

[aos15247-bib-0042] Dawson, S.R. , Mallen, C.D. , Gouldstone, M.B. , Yarham, R. & Mansell, G. (2014) The prevalence of anxiety and depression in people with age‐related macular degeneration: a systematic review of observational study data. BMC Ophthalmology, 14, 78. 24923726 10.1186/1471-2415-14-78 PMC4094542

[aos15247-bib-0043] Day, S. , Acquah, K. , Lee, P.P. , Mruthyunjaya, P. & Sloan, F.A. (2011) Medicare costs for neovascular age‐related macular degeneration, 1994‐2007. American Journal of Ophthalmology, 152, 1014–1020. 21843875 10.1016/j.ajo.2011.05.008 PMC3219793

[aos15247-bib-0044] DiDonato, J.A. , Mercurio, F. & Karin, M. (2012) NF‐κB and the link between inflammation and cancer. Immunological Reviews, 246, 379–400. 22435567 10.1111/j.1600-065X.2012.01099.x

[aos15247-bib-0045] Dimberg, A. (2010) Chemokines in angiogenesis. Berlin, Heidelberg: Springer, pp. 59–80.

[aos15247-bib-0046] Drew, W. , Wilson, D.V. & Sapey, E. (2018) Inflammation and neutrophil immunosenescence in health and disease: targeted treatments to improve clinical outcomes in the elderly. Experimental Gerontology, 105, 70–77. 29288715 10.1016/j.exger.2017.12.020

[aos15247-bib-0047] Ebrahimi, K.B. , Fijalkowski, N. , Cano, M. & Handa, J.T. (2013) Decreased membrane complement regulators in the retinal pigmented epithelium contributes to age‐related macular degeneration. Journal of Pathology, 229, 729–742. 23097248 10.1002/path.4128 PMC3836183

[aos15247-bib-0048] Ehlers, S. & Kaufmann, S.H.E. (2010) Infection, inflammation, and chronic diseases: consequences of a modern lifestyle. Trends in Immunology, 31, 184–190. 20399709 10.1016/j.it.2010.02.003

[aos15247-bib-0049] Ellulu, M.S. , Patimah, I. , Khaza'ai, H. , Rahmat, A. & Abed, Y. (2017) Obesity and inflammation: the linking mechanism and the complications. Archives of Medical Science, 13, 851–863. 28721154 10.5114/aoms.2016.58928 PMC5507106

[aos15247-bib-0050] Espinosa‐Heidmann, D.G. , Suner, I.J. , Hernandez, E.P. , Monroy, D. , Csaky, K.G. & Cousins, S.W. (2003) Macrophage depletion diminishes lesion size and severity in experimental choroidal neovascularization. Investigative Ophthalmology & Visual Science, 44, 3586–3592. 12882811 10.1167/iovs.03-0038

[aos15247-bib-0051] Ezzat, M.‐K. , Hann, C.R. , Vuk‐Pavlovic, S. & Pulido, J.S. (2008) Immune cells in the human choroid. The British Journal of Ophthalmology, 92, 976–980. 18577650 10.1136/bjo.2007.129742

[aos15247-bib-0052] Faber, C. , Singh, A. , Krüger Falk, M. , Juel, H.B. , Sørensen, T.L. & Nissen, M.H. (2013) Age‐related macular degeneration is associated with increased proportion of CD56+T cells in peripheral blood. Ophthalmology, 120, 2310–2316. 23747161 10.1016/j.ophtha.2013.04.014

[aos15247-bib-0053] Falk, M.K. , Singh, A. , Faber, C. , Nissen, M.H. , Hviid, T. & Sørensen, T.L. (2014) Dysregulation of CXCR3 expression on peripheral blood leukocytes in patients with neovascular age‐related macular degeneration. Investigative Ophthalmology & Visual Science, 55, 4050. 24812555 10.1167/iovs.14-14107

[aos15247-bib-0054] Fan, Y. , Mao, R. & Yang, J. (2013) NF‐κB and STAT3 signaling pathways collaboratively link inflammation to cancer. Protein Cell, 4, 176–185. 23483479 10.1007/s13238-013-2084-3 PMC4875500

[aos15247-bib-0055] Ferris, F.L. , Wilkinson, C.P. , Bird, A. , Chakravarthy, U. , Chew, E. , Csaky, K. et al. (2013) Clinical classification of age‐related macular degeneration. Ophthalmology, 120, 844–851. 23332590 10.1016/j.ophtha.2012.10.036 PMC11551519

[aos15247-bib-0056] Fisher, D.A.C. , Fowles, J.S. , Zhou, A. & Oh, S.T. (2021) Inflammatory pathophysiology as a contributor to myeloproliferative neoplasms. Frontiers in Immunology, 12, 683401. 34140953 10.3389/fimmu.2021.683401 PMC8204249

[aos15247-bib-0057] Fleckenstein, M. , Keenan, T.D.L. , Guymer, R.H. , Chakravarthy, U. , Schmitz‐Valckenberg, S. , Klaver, C.C. et al. (2021) Age‐related macular degeneration. Nature Reviews Disease Primers, 7, 31. 10.1038/s41572-021-00265-2 PMC12878645 33958600

[aos15247-bib-0058] Fleckenstein, M. , Mitchell, P. , Freund, K.B. , Sadda, S.V. , Holz, F.G. , Brittain, C. et al. (2018) The progression of geographic atrophy secondary to age‐related macular degeneration. Ophthalmology, 125, 369–390. 29110945 10.1016/j.ophtha.2017.08.038

[aos15247-bib-0059] Folkman, J. (1995) Angiogenesis in cancer, vascular, rheumatoid and other disease. Nature Medicine, 1, 27–30. 10.1038/nm0195-27 7584949

[aos15247-bib-0060] Franceschi, C. , Bonafè, M. , Valensin, S. , Olivieri, F. , De Luca, M. , Ottaviani, E. et al. (2006) Inflamm‐aging: an evolutionary perspective on immunosenescence. Annals of the New York Academy of Sciences, 908, 244–254. 10.1111/j.1749-6632.2000.tb06651.x 10911963

[aos15247-bib-0061] Frederiksen, H. , Farkas, D.K. , Christiansen, C.F. , Hasselbalch, H.C. & Sørensen, H.T. (2011) Chronic myeloproliferative neoplasms and subsequent cancer risk: a Danish population‐based cohort study. Blood, 118, 6515–6520. 22039256 10.1182/blood-2011-04-348755

[aos15247-bib-0062] Fritsch, R.D. , Shen, X. , Sims, G.P. , Hathcock, K.S. , Hodes, R.J. & Lipsky, P.E. (2005) Stepwise differentiation of CD4 memory T cells defined by expression of CCR7 and CD27. Journal of Immunology, 175, 6489–6497. 10.4049/jimmunol.175.10.6489 16272303

[aos15247-bib-0063] Fulop, T. , Larbi, A. , Dupuis, G. , Le Page, A. , Frost, E.H. , Cohen, A.A. et al. (2018) Immunosenescence and inflamm‐aging as two sides of the same coin: friends or foes? Frontiers in Immunology, 8, 1960. 29375577 10.3389/fimmu.2017.01960 PMC5767595

[aos15247-bib-0064] GBD 2019 Blindness and Vision Impairment Collaborators & Vision Loss Expert Group of the Global Burden of Disease Study . (2021) Causes of blindness and vision impairment in 2020 and trends over 30 years, and prevalence of avoidable blindness in relation to VISION 2020: the Right to Sight: an analysis for the Global Burden of Disease Study. Lancet Global Health, 9, e144–e160. 33275949 10.1016/S2214-109X(20)30489-7 PMC7820391

[aos15247-bib-0065] Germolec, D.R. , Shipkowski, K.A. , Frawley, R.P. & Evans, E. (2018) Markers of inflammation. New York, NY: Humana Press, pp. 57–79. 10.1007/978-1-4939-8549-4_5 29882133

[aos15247-bib-0066] Ghosh, S. , Padmanabhan, A. , Vaidya, T. , Watson, A.M. , Bhutto, I.A. , Hose, S. et al. (2019) Neutrophils homing into the retina trigger pathology in early age‐related macular degeneration. Communications Biology, 21(2), 1–17. 10.1038/s42003-019-0588-y PMC6754381 31552301

[aos15247-bib-0067] Goronzy, J.J. , Fang, F. , Cavanagh, M.M. , Qi, Q. & Weyand, C.M. (2015) Naive T cell maintenance and function in human aging. Journal of Immunology, 194, 4073–4080. 10.4049/jimmunol.1500046 PMC4452284 25888703

[aos15247-bib-0068] Groom, J.R. & Luster, A.D. (2011) CXCR3 ligands: redundant, collaborative and antagonistic functions. Immunology and Cell Biology, 89, 207–215. 21221121 10.1038/icb.2010.158 PMC3863330

[aos15247-bib-0069] Guillonneau, X. , Eandi, C.M. , Paques, M. , Sahel, J.‐A. , Sapieha, P. & Sennlaub, F. (2017) On phagocytes and macular degeneration. Progress in Retinal and Eye Research, 61, 98–128. 28602950 10.1016/j.preteyeres.2017.06.002

[aos15247-bib-0070] Gupta, N. , Brown, K.E. & Milam, A.H. (2003) Activated microglia in human retinitis pigmentosa, late‐onset retinal degeneration, and age‐related macular degeneration. Experimental Eye Research, 76, 463–471. 12634111 10.1016/s0014-4835(02)00332-9

[aos15247-bib-0071] Hansson, G.K. (2009) Inflammatory mechanisms in atherosclerosis. Journal of Thrombosis and Haemostasis, 7, 328–331. 19630827 10.1111/j.1538-7836.2009.03416.x

[aos15247-bib-0072] Hansson, G.K. , Robertson, A.K.L. & Söderberg‐Nauclér, C. (2006) Inflammation and atherosclerosis. Annual Review of Pathology, 1, 297–329. 10.1146/annurev.pathol.1.110304.100100 18039117

[aos15247-bib-0073] Hasselbalch, H.C. (2012) Perspectives on chronic inflammation in essential thrombocythemia, polycythemia vera, and myelofibrosis: is chronic inflammation a trigger and driver of clonal evolution and development of accelerated atherosclerosis and second cancer? Blood, 119, 3219–3225. 22318201 10.1182/blood-2011-11-394775

[aos15247-bib-0074] Hasselbalch, H.C. (2013) Chronic inflammation as a promotor of mutagenesis in essential thrombocythemia, polycythemia vera and myelofibrosis. A human inflammation model for cancer development? Leukemia Research, 37, 214–220. 23174192 10.1016/j.leukres.2012.10.020

[aos15247-bib-0075] Hasselbalch, H.C. (2015) Perspectives on the increased risk of second cancer in patients with essential thrombocythemia, polycythemia vera and myelofibrosis. European Journal of Haematology, 94, 96–98. 25689636 10.1111/ejh.12437

[aos15247-bib-0076] Hasselbalch, H.C. & Bjørn, M.E. (2015) MPNs as inflammatory diseases: The evidence, consequences, and perspectives. Mediators of Inflammation, 2015, 1–16. 10.1155/2015/102476 PMC4641200 26604428

[aos15247-bib-0077] Hasselbalch, H.C. & Holmström, M.O. (2019) Perspectives on interferon‐alpha in the treatment of polycythemia vera and related myeloproliferative neoplasms: minimal residual disease and cure? Seminars in Immunopathology, 41, 5–19. 30203226 10.1007/s00281-018-0700-2 PMC6323070

[aos15247-bib-0078] Hasselbalch, H.C. , Skov, V. , Kjær, L. , Sørensen, T.L. , Ellervik, C. & Wienecke, T. (2020) Myeloproliferative blood cancers as a human neuroinflammation model for development of Alzheimer's disease: evidences and perspectives. Journal of Neuroinflammation, 17, 248. 32829706 10.1186/s12974-020-01877-3 PMC7444051

[aos15247-bib-0079] Heesterbeek, T.J. , Lorés‐Motta, L. , Hoyng, C.B. , Lechanteur, Y.T.E. & den Hollander, A.I. (2020) Risk factors for progression of age‐related macular degeneration. Ophthalmic & Physiological Optics, 40, 140–170. 32100327 10.1111/opo.12675 PMC7155063

[aos15247-bib-0080] Hoermann, G. , Greiner, G. & Valent, P. (2015) Cytokine regulation of microenvironmental cells in myeloproliferative neoplasms. Mediators of Inflammation, 2015, 1–17. 10.1155/2015/869242 PMC4620237 26543328

[aos15247-bib-0081] Holmes, C. (2013) Review: systemic inflammation and Alzheimer's disease. Neuropathology and Applied Neurobiology, 39, 51–68. 23046210 10.1111/j.1365-2990.2012.01307.x

[aos15247-bib-0082] Hong, T. , Tan, A.G. , Mitchell, P. & Wang, J.J. (2011) A review and meta‐analysis of the association between C‐reactive protein and age‐related macular degeneration. Survey of Ophthalmology, 56, 184–194. 21420705 10.1016/j.survophthal.2010.08.007

[aos15247-bib-0083] Hou, Y. , Dan, X. , Babbar, M. , Wei, Y. , Hasselbalch, S.G. , Croteau, D.L. et al. (2019) Ageing as a risk factor for neurodegenerative disease. Nature Reviews. Neurology, 15, 565–581. 31501588 10.1038/s41582-019-0244-7

[aos15247-bib-0084] Hughes, C.E. & Nibbs, R.J.B. (2018) A guide to chemokines and their receptors. The FEBS Journal, 285, 2944–2971. 29637711 10.1111/febs.14466 PMC6120486

[aos15247-bib-0085] Kaarniranta, K. , Sinha, D. , Blasiak, J. , Kauppinen, A. , Veréb, Z. , Salminen, A. et al. (2013) Autophagy and heterophagy dysregulation leads to retinal pigment epithelium dysfunction and development of age‐related macular degeneration. Autophagy, 9, 973. 23590900 10.4161/auto.24546 PMC3722332

[aos15247-bib-0086] Karin, M. & Greten, F.R. (2005) NF‐κB: linking inflammation and immunity to cancer development and progression. Nature Reviews. Immunology, 5, 749–759. 10.1038/nri1703 16175180

[aos15247-bib-0087] Kauppinen, A. , Paterno, J.J. , Blasiak, J. , Salminen, A. & Kaarniranta, K. (2016) Inflammation and its role in age‐related macular degeneration. Cellular and Molecular Life Sciences, 73, 1765–1786. 26852158 10.1007/s00018-016-2147-8 PMC4819943

[aos15247-bib-0088] Kersten, E. , Paun, C.C. , Schellevis, R.L. , Hoyng, C.B. , Delcourt, C. , Lengyel, I. et al. (2018) Systemic and ocular fluid compounds as potential biomarkers in age‐related macular degeneration. Survey of Ophthalmology, 63, 9–39. 28522341 10.1016/j.survophthal.2017.05.003

[aos15247-bib-0089] Kikuchi, M. , Nakamura, M. , Ishikawa, K. , Suzuki, T. , Nishihara, H. , Yamakoshi, T. et al. (2007) Elevated C‐reactive protein levels in patients with polypoidal choroidal vasculopathy and patients with neovascular age‐related macular degeneration. Ophthalmology, 114, 1722–1727. 17400294 10.1016/j.ophtha.2006.12.021

[aos15247-bib-0090] Kim, S.‐Y. , Bae, S.H. , Bang, S.‐M. , Eom, K.S. , Hong, J. , Jang, S. et al. (2021) The 2020 revision of the guidelines for the management of myeloproliferative neoplasms. Korean Journal of Internal Medicine, 36, 45–62. 33147902 10.3904/kjim.2020.319 PMC7820646

[aos15247-bib-0091] Klein, R. , Klein, B.E. & Linton, K.L. (1992) Prevalence of age‐related maculopathy. The Beaver Dam Eye Study. Ophthalmology, 99, 933–943. 1630784 10.1016/s0161-6420(92)31871-8

[aos15247-bib-0092] Kolb, H. (2007) Facts and figures concerning the human retina. In: Kolb, H. , Fernandez, E. & Nelson, R. (Eds.) Webvision: The organization of the retina and visual system. Salt Lake City, UT: University of Utah Health Sciences Center. 21413409

[aos15247-bib-0093] Kristinsson, S.Y. , Landgren, O. , Samuelsson, J. , Björkholm, M. & Goldin, L.R. (2010) Autoimmunity and the risk of myeloproliferative neoplasms. Haematologica, 95, 1216–1220. 20053870 10.3324/haematol.2009.020412 PMC2895049

[aos15247-bib-0094] Krogh Nielsen, M. , Subhi, Y. , Rue Molbech, C. , Nilsson, L.L. , Nissen, M.H. & Sørensen, T.L. (2019) Imbalances in tissue inhibitors of metalloproteinases differentiate choroidal neovascularization from geographic atrophy. Acta Ophthalmologica, 97, 84–90. 30288950 10.1111/aos.13894

[aos15247-bib-0095] Lad, E.M. , Cousins, S.W. , Van Arnam, J.S. & Proia, A.D. (2015) Abundance of infiltrating CD163+ cells in the retina of postmortem eyes with dry and neovascular age‐related macular degeneration. Graefe's Archive for Clinical and Experimental Ophthalmology, 253, 1941–1945. 10.1007/s00417-015-3094-z PMC5203931 26148801

[aos15247-bib-0096] Lambert, N.G. , ElShelmani, H. , Singh, M.K. , Mansergh, F.C. , Wride, M.A. , Padilla, M. et al. (2016) Risk factors and biomarkers of age‐related macular degeneration. Progress in Retinal and Eye Research, 54, 64–102. 27156982 10.1016/j.preteyeres.2016.04.003 PMC4992630

[aos15247-bib-0097] Larsen, T.S. , Pallisgaard, N. , Møller, M.B. & Hasselbalch, H.C. (2007) The JAK2 V617F allele burden in essential thrombocythemia, polycythemia vera and primary myelofibrosis – impact on disease phenotype. European Journal of Haematology, 79, 508–515. 17961178 10.1111/j.1600-0609.2007.00960.x

[aos15247-bib-0098] Lathe, R. , Sapronova, A. & Kotelevtsev, Y. (2014) Atherosclerosis and Alzheimer‐‐diseases with a common cause? Inflammation, oxysterols, vasculature. BMC Geriatrics, 14, 36. 24656052 10.1186/1471-2318-14-36 PMC3994432

[aos15247-bib-0099] Lechner, J. , Chen, M. , Hogg, R.E. , Toth, L. , Silvestri, G. , Chakravarthy, U. et al. (2016) Higher plasma levels of complement C3a, C4a and C5a increase the risk of subretinal fibrosis in neovascular age‐related macular degeneration. Immunity & Ageing, 13, 4. 26884800 10.1186/s12979-016-0060-5 PMC4754842

[aos15247-bib-0100] Lemster, B.H. , Michel, J.J. , Montag, D.T. , Paat, J.J. , Studenski, S.A. , Newman, A.B. et al. (2008) Induction of CD56 and TCR‐independent activation of T cells with aging. Journal of Immunology, 180, 1979–1990. 10.4049/jimmunol.180.3.1979 18209097

[aos15247-bib-0101] Lentsch, A.B. & Ward, P.A. (2000) Regulation of inflammatory vascular damage. The Journal of Pathology, 190, 343–348. 10685068 10.1002/(SICI)1096-9896(200002)190:3<343::AID-PATH522>3.0.CO;2-M

[aos15247-bib-0102] Liisborg, C. , Hasselbalch, H.C. & Sørensen, T.L. (2020) Ocular manifestations in patients with philadelphia‐negative myeloproliferative neoplasms. Cancers, 12, 573. 32121664 10.3390/cancers12030573 PMC7139696

[aos15247-bib-0103] Ling, M. & Murali, M. (2019) Analysis of the complement system in the Clinical Immunology Laboratory. Clinics in Laboratory Medicine, 39, 579–590. 31668271 10.1016/j.cll.2019.07.006

[aos15247-bib-0104] Liukkonen, M. , Paterno, J. , Koskela, A. & Kaarniranta, K. (2022) Serum endothelin 1 and interleukin 8 are elevated in patients with active wet age‐related macular degeneration without a clear serological link to epithelial‐mesenchymal transition: a Finnish cohort. Acta Ophthalmologica, 100(S267): 1755. 10.1111/j.1755-3768.2022.001 34699684

[aos15247-bib-0105] López‐Otín, C. , Blasco, M.A. , Partridge, L. , Serrano, M. & Kroemer, G. (2013) The hallmarks of aging. Cell, 153, 1194–1217. 23746838 10.1016/j.cell.2013.05.039 PMC3836174

[aos15247-bib-0106] Luster, A.D. (1998) Chemokines — chemotactic cytokines that mediate inflammation. The New England Journal of Medicine, 338, 436–445. 9459648 10.1056/NEJM199802123380706

[aos15247-bib-0107] Lynch, A.M. , Mandava, N. , Patnaik, J.L. , Frazer‐Abel, A.A. , Wagner, B.D. , Palestine, A.G. et al. (2020a) Systemic activation of the complement system in patients with advanced age‐related macular degeneration. European Journal of Ophthalmology, 30, 1061–1068. 31203676 10.1177/1120672119857896

[aos15247-bib-0108] Lynch, A.M. , Palestine, A.G. , Wagner, B.D. , Patnaik, J.L. , Frazier‐Abel, A.A. , Mathias, M.T. et al. (2020b) Complement factors and reticular pseudodrusen in intermediate age‐related macular degeneration staged by multimodal imaging. BMJ Open Ophthalmology, 5, e000361. 10.1136/bmjophth-2019-000361 PMC7254108 32509962

[aos15247-bib-0109] Lynch, A.M. , Wagner, B.D. , Palestine, A.G. , Janjic, N. , Patnaik, J.L. , Mathias, M.T. et al. (2020c) Plasma biomarkers of reticular pseudodrusen and the risk of progression to advanced age‐related macular degeneration. Translational Vision Science & Technology, 9, 12–12. 10.1167/tvst.9.10.12 PMC7488626 32974084

[aos15247-bib-0110] Machalińska, A. , Dziedziejko, V. , Mozolewska‐Piotrowska, K. , Karczewicz, D. , Wiszniewska, B. & Machaliński, B. (2009) Elevated plasma levels of C3a complement compound in the exudative form of age‐related macular degeneration. Ophthalmic Research, 42, 54–59. 19478542 10.1159/000219686

[aos15247-bib-0111] Martínez‐Alberquilla, I. , Gasull, X. , Pérez‐Luna, P. , Seco‐Mera, R. , Ruiz‐Alcocer, J. & Crooke, A. (2022) Neutrophils and neutrophil extracellular trap components: emerging biomarkers and therapeutic targets for age‐related eye diseases. Ageing Research Reviews, 74, 101553. 34971794 10.1016/j.arr.2021.101553

[aos15247-bib-0112] Mathieu, P. , Lemieux, I. & Després, J.‐P. (2010) Obesity, inflammation, and cardiovascular risk. Clinical Pharmacology & Therapeutics, 87, 407–416. 20200516 10.1038/clpt.2009.311

[aos15247-bib-0113] McGeer, P.L. & McGeer, E.G. (2004) Inflammation and the degenerative diseases of aging. Annals of the New York Academy of Sciences, 1035, 104–116. 15681803 10.1196/annals.1332.007

[aos15247-bib-0114] McGuinness, M.B. , Simpson, J.A. & Finger, R.P. (2018) Analysis of the association between physical activity and age‐related macular degeneration. JAMA Ophthalmol, 136, 139–140. 29242921 10.1001/jamaophthalmol.2017.4782

[aos15247-bib-0115] McMullin, M.F. & Anderson, L.A. (2020) Aetiology of myeloproliferative neoplasms. Cancers, 12(7): 1810. 32640679 10.3390/cancers12071810 PMC7408762

[aos15247-bib-0116] Medinger, M. & Passweg, J. (2014) Angiogenesis in myeloproliferative neoplasms, new markers and future directions. Memo, 7, 206–210. 25544863 10.1007/s12254-014-0142-z PMC4274371

[aos15247-bib-0117] Medinger, M. , Skoda, R. , Gratwohl, A. , Theocharides, A. , Buser, A. , Heim, D. et al. (2009) Angiogenesis and vascular endothelial growth factor‐/receptor expression in myeloproliferative neoplasms: correlation with clinical parameters and *JAK2‐V617F* mutational status. British Journal of Haematology, 146, 150–157. 19466975 10.1111/j.1365-2141.2009.07726.x

[aos15247-bib-0118] Medzhitov, R. (2008) Origin and physiological roles of inflammation. Nature, 454(7203): 428–435. 18650913 10.1038/nature07201

[aos15247-bib-0119] Mendez Luque, L.F. , Blackmon, A.L. , Ramanathan, G. & Fleischman, A.G. (2019) Key role of inflammation in myeloproliferative neoplasms: instigator of disease initiation, progression and symptoms. Current Hematologic Malignancy Reports, 14, 145–153. 31119475 10.1007/s11899-019-00508-w PMC7746200

[aos15247-bib-0120] Mikkelsen, S.U. , Kjaer, L. , Bjørn, M.E. , Knudsen, T.A. , Sørensen, A.L. , Andersen, C.B.L. et al. (2018) Safety and efficacy of combination therapy of interferon‐α2 and ruxolitinib in polycythemia vera and myelofibrosis. Cancer Medicine, 7, 3571–3581. 29932310 10.1002/cam4.1619 PMC6089176

[aos15247-bib-0121] Mitchell, P. , Smith, W. , Attebo, K. & Wang, J.J. (1995) Prevalence of age‐related maculopathy in Australia: the Blue Mountains Eye Study. Ophthalmology, 102, 1450–1460. 9097791 10.1016/s0161-6420(95)30846-9

[aos15247-bib-0122] Mitta, V.P. , Christen, W.G. , Glynn, R.J. , Semba, R.D. , Ridker, P.M. , Rimm, E.B. et al. (2013) C‐reactive protein and the incidence of macular degeneration: pooled analysis of 5 cohorts. JAMA Ophthalmology, 131, 507–513. 23392454 10.1001/jamaophthalmol.2013.2303 PMC3625501

[aos15247-bib-0123] Modjtahedi, B.S. , Fong, D.S. , Jorgenson, E. , Van Den Eeden, S.K. , Quinn, V. & Slezak, J.M. (2018) The relationship between nonsteroidal anti‐inflammatory drug use and age‐related macular degeneration. American Journal of Ophthalmology, 188, 111–122. 29360460 10.1016/j.ajo.2018.01.012

[aos15247-bib-0124] Moro‐García, M.A. , Alonso‐Arias, R. & Lopez‐Larrea, C. (2013) When aging reaches CD4+ T‐cells: phenotypic and functional changes. Frontiers in Immunology, 4, 107. 23675374 10.3389/fimmu.2013.00107 PMC3650461

[aos15247-bib-0125] Nassar, K. , Grisanti, S. , Elfar, E. , Lüke, J. , Lüke, M. & Grisanti, S. (2015) Serum cytokines as biomarkers for age‐related macular degeneration. Graefes Arch Clin Exp Ophthalmol, 253, 699–704. 25056526 10.1007/s00417-014-2738-8

[aos15247-bib-0126] Netea, M.G. , Balkwill, F. , Chonchol, M. , Cominelli, F. , Donath, M.Y. , Giamarellos‐Bourboulis, E.J. et al. (2017) A guiding map for inflammation. Nature Immunology, 18, 826–831. 28722720 10.1038/ni.3790 PMC5939996

[aos15247-bib-0127] Niazi, S. , Krogh Nielsen, M. , Sørensen, T.L. & Subhi, Y. (2019) Neutrophil‐to‐lymphocyte ratio in age‐related macular degeneration: a systematic review and meta‐analysis. Acta Ophthalmologica, 97, 558–566. 30811869 10.1111/aos.14072

[aos15247-bib-0128] Niccoli, T. & Partridge, L. (2012) Ageing as a risk factor for disease. Current Biology, 22, R741–R752. 22975005 10.1016/j.cub.2012.07.024

[aos15247-bib-0129] Nowak, J.Z. (2014) Aspirin and age‐related macular degeneration: positives versus negatives. Expert Opinion on Drug Safety, 13, 687–690. 24783984 10.1517/14740338.2014.915939

[aos15247-bib-0130] Park, D.H. , Connor, K.M. & Lambris, J.D. (2019) The challenges and promise of complement therapeutics for ocular diseases. Frontiers in Immunology, 10, 1007. 31156618 10.3389/fimmu.2019.01007 PMC6529562

[aos15247-bib-0131] Pawelec, G. (2017) Age and immunity: What is ‘immunosenescence’? Experimental Gerontology, 105, 4–9. 29111233 10.1016/j.exger.2017.10.024

[aos15247-bib-0132] Penfold, P. , Killingsworth, M. & Fraco, S.S. (1984) An ultrastructural study of the role of leucocytes and fibroblasts in the breakdown of Bruch's membrane. Australian Journal of Ophthalmology, 12, 23–31. 6732655

[aos15247-bib-0133] Penfold, P.L. , Killingsworth, M.C. & Sarks, S.H. (1986) Senile macular degeneration. The involvement of giant cells in atrophy of the retinal pigment epithelium. Investigative Ophthalmology & Visual Science, 27, 364–371. 3949464

[aos15247-bib-0134] Peniket, A.J. (2002) The myeloproliferative disorders. CPD Bulletin Cellular Pathology, 4, 35–38.

[aos15247-bib-0135] Pereira, B.I. , De Maeyer, R.P.H. , Covre, L.P. , Nehar‐Belaid, D. , Lanna, A. , Ward, S. et al. (2020) Sestrins induce natural killer function in senescent‐like CD8+ T cells. Nature Immunology, 21, 684–694. 32231301 10.1038/s41590-020-0643-3 PMC10249464

[aos15247-bib-0136] Pittet, M.J. , Speiser, D.E. , Valmori, D. , Cerottini, J.C. & Romero, P. (2000) Cutting edge: cytolytic effector function in human circulating CD8+ T cells closely correlates with CD56 surface expression. Journal of Immunology, 164, 1148–1152. 10.4049/jimmunol.164.3.1148 10640724

[aos15247-bib-0137] Reisner, S.A. , Rinkevich, D. , Markiewicz, W. , Tatarsky, I. & Brenner, B. (1992) Cardiac involvement in patients with myeloproliferative disorders. The American Journal of Medicine, 93, 498–504. 1442851 10.1016/0002-9343(92)90576-w

[aos15247-bib-0138] Reynolds, R. , Hartnett, M.E. , Atkinson, J.P. , Giclas, P.C. , Rosner, B. & Seddon, J.M. (2009) Plasma complement components and activation fragments: associations with age‐related macular degeneration genotypes and phenotypes. Investigative Ophthalmology & Visual Science, 50, 5818. 19661236 10.1167/iovs.09-3928 PMC2826794

[aos15247-bib-0139] Ricci, F. , Bandello, F. , Navarra, P. , Staurenghi, G. , Stumpp, M. & Zarbin, M. (2020) Neovascular age‐related macular degeneration: therapeutic management and new‐upcoming approaches. International Journal of Molecular Sciences, 21, 1–40. 10.3390/ijms21218242 PMC7662479 33153227

[aos15247-bib-0140] Risau, W. (1997) Mechanisms of angiogenesis. Nature, 386, 671–674. 9109485 10.1038/386671a0

[aos15247-bib-0141] Ristau, T. , Paun, C. , Ersoy, L. , Hahn, M. , Lechanteur, Y. , Hoyng, C. et al. (2014) Impact of the common genetic associations of age‐related macular degeneration upon systemic complement component C3d levels. PLoS One, 9, e93459. 24675670 10.1371/journal.pone.0093459 PMC3968152

[aos15247-bib-0142] Roizenblatt, M. , Naranjit, N. , Maia, M. & Gehlbach, P.L. (2018) The question of a role for statins in age‐related macular degeneration. International Journal of Molecular Sciences, 19, 3688. 30469381 10.3390/ijms19113688 PMC6274767

[aos15247-bib-0143] Romagnani, P. , Lasagni, L. , Annunziato, F. , Serio, M. & Romagnani, S. (2004) CXC chemokines: the regulatory link between inflammation and angiogenesis. Trends in Immunology, 25, 201–209. 15039047 10.1016/j.it.2004.02.006

[aos15247-bib-0144] Rozing, M.P. , Durhuus, J.A. , Krogh Nielsen, M. , Subhi, Y. , Kirkwood, T.B. , Westendorp, R.G. et al. (2020) Age‐related macular degeneration: a two‐level model hypothesis. Progress in Retinal and Eye Research, 76, 100825. 31899290 10.1016/j.preteyeres.2019.100825

[aos15247-bib-0145] Sakurai, E. , Anand, A. , Ambati, B.K. , Van Rooijen, N. & Ambati, J. (2003) Macrophage depletion inhibits experimental choroidal neovascularization. Investigative Ophthalmology & Visual Science, 44, 3578–3585. 12882810 10.1167/iovs.03-0097

[aos15247-bib-0146] Schnabolk, G. (2019) Systemic inflammatory disease and AMD comorbidity. Advances in Experimental Medicine and Biology, 1185, 27–31. 31884584 10.1007/978-3-030-27378-1_5

[aos15247-bib-0147] Schnabolk, G. , Rohrer, B. , Simpson, K.N. & Schna‐Bolk, G. (2019) Clinical and epidemiologic research increased nonexudative age‐related macular degeneration diagnosis among medicare beneficiaries with rheumatoid arthritis. Investigative Ophthalmology & Visual Science, 60, 3520–3526. 31412111 10.1167/iovs.18-26444 PMC6694737

[aos15247-bib-0148] Scholl, H.P.N. , Issa, P.C. , Walier, M. , Janzer, S. , Pollok‐Kopp, B. , Börncke, F. et al. (2008) Systemic complement activation in age‐related macular degeneration. PLoS One, 3, e2593. 18596911 10.1371/journal.pone.0002593 PMC2440421

[aos15247-bib-0149] Seddon, J.M. , Gensler, G. , Milton, R.C. , Klein, M.L. & Rifai, N. (2004) Association between C‐reactive protein and age‐related macular degeneration. JAMA, 291, 704–710. 14871913 10.1001/jama.291.6.704

[aos15247-bib-0150] Sedeh, F.B. , Scott, D.A.R. , Subhi, Y. & Sørensen, T.L. (2017) Prevalence of neovascular age‐related macular degeneration and geographic atrophy in Denmark. Danish Medical Journal, 64, A5422. 29115208

[aos15247-bib-0151] Sennlaub, F. , Auvynet, C. , Calippe, B. , Lavalette, S. , Poupel, L. , Hu, S.J. et al. (2013) CCR 2 + monocytes infiltrate atrophic lesions in age‐related macular disease and mediate photoreceptor degeneration in experimental subretinal inflammation in Cx3cr1 deficient mice. EMBO Molecular Medicine, 5, 1775–1793. 24142887 10.1002/emmm.201302692 PMC3840491

[aos15247-bib-0152] Shallis, R. , Zeidan, A. , Wang, R. & Podoltsev, N. (2021) Epidemiology of the philadelphia chromosome‐negative classical myeloproliferative neoplasms. Hematology/Oncology Clinics of North America, 35, 177–189. 33641862 10.1016/j.hoc.2020.11.005

[aos15247-bib-0153] Shallis, R.M. , Wang, R. , Davidoff, A. , Ma, X. , Podoltsev, N.A. & Zeidan, A.M. (2020) Epidemiology of the classical myeloproliferative neoplasms: The four corners of an expansive and complex map. Blood Reviews, 42, 100706. 32517877 10.1016/j.blre.2020.100706

[aos15247-bib-0154] Singh, A. , Faber, C. , Falk, M. , Nissen, M.H. , Hviid, T.V.F. & Sørensen, T.L. (2012) Altered expression of CD46 and CD59 on leukocytes in neovascular age‐related macular degeneration. American Journal of Ophthalmology, 154, 193–199.e2. 22541656 10.1016/j.ajo.2012.01.036

[aos15247-bib-0155] Singh, A. , Subhi, Y. , Nielsen, M.K. , Falk, M.K. , Matzen, S.M.H. , Sellebjerg, F. et al. (2017) Systemic frequencies of T helper 1 and T helper 17 cells in patients with age‐related macular degeneration: A case‐control study. Scientific Reports, 7, 1–9. 28377586 10.1038/s41598-017-00741-4 PMC5429667

[aos15247-bib-0156] Sivaprasad, S. , Adewoyin, T. , Bailey, T.A. , Dandekar, S.S. , Jenkins, S. , Webster, A.R. et al. (2007) Estimation of systemic complement C3 activity in age‐related macular degeneration. Archives of Ophthalmology, 125, 515–519. 17420372 10.1001/archopht.125.4.515

[aos15247-bib-0157] Smailhodzic, D. , Klaver, C.C.W. , Klevering, B.J. , Boon, C.J. , Groenewoud, J.M. , Kirchhof, B. et al. (2012) Risk alleles in CFH and ARMS2 are independently associated with systemic complement activation in age‐related macular degeneration. Ophthalmology, 119, 339–346. 22133792 10.1016/j.ophtha.2011.07.056

[aos15247-bib-0158] Soulas, C. , Donahue, R.E. , Dunbar, C.E. , Persons, D.A. , Alvarez, X. & Williams, K.C. (2009) Genetically modified CD34+ hematopoietic stem cells contribute to turnover of brain perivascular macrophages in long‐term repopulated primates. The American Journal of Pathology, 174, 1808. 19349370 10.2353/ajpath.2009.081010 PMC2671269

[aos15247-bib-0159] Spaide, R.F. , Ooto, S. & Curcio, C.A. (2018) Subretinal drusenoid deposits AKA pseudodrusen. Survey of Ophthalmology, 63, 782–815. 29859199 10.1016/j.survophthal.2018.05.005

[aos15247-bib-0160] Spivak, J.L. (2017) Myeloproliferative neoplasms. New England Journal of Medicine, 376, 2168–2181. 28564565 10.1056/NEJMra1406186

[aos15247-bib-0161] Stoll, G. & Bendszus, M. (2006) Inflammation and atherosclerosis: novel insights into plaque formation and destabilization. Stroke, 37, 1923–1932. 16741184 10.1161/01.STR.0000226901.34927.10

[aos15247-bib-0162] Subhi, Y. , Nielsen, M.K. , Molbech, C.R. , Oishi, A. , Singh, A. , Nissen, M.H. et al. (2017) T‐cell differentiation and CD56+ levels in polypoidal choroidal vasculopathy and neovascular age‐related macular degeneration. Aging, 9, 2436–2452. 29165313 10.18632/aging.101329 PMC5723695

[aos15247-bib-0163] Taylor, D.J. , Hobby, A.E. , Binns, A.M. & Crabb, D.P. (2016) How does age‐related macular degeneration affect real‐world visual ability and quality of life? A systematic review. BMJ Open, 6, e011504. 10.1136/bmjopen-2016-011504 PMC5168634 27913556

[aos15247-bib-0164] Tefferi, A. & Pardanani, A. (2015) Myeloproliferative neoplasms. JAMA Oncology, 1, 97. 26182311 10.1001/jamaoncol.2015.89

[aos15247-bib-0165] Tefferi, A. , Rumi, E. , Finazzi, G. , Gisslinger, H. , Vannucchi, A.M. , Rodeghiero, F. et al. (2013) Survival and prognosis among 1545 patients with contemporary polycythemia vera: an international study. Leukemia, 27, 1874–1881. 23739289 10.1038/leu.2013.163 PMC3768558

[aos15247-bib-0166] Tsutsumi, C. , Sonoda, K.‐H. , Egashira, K. , Qiao, H. , Hisatomi, T. , Nakao, S. et al. (2003) The critical role of ocular‐infiltrating macrophages in the development of choroidal neovascularization. Journal of Leukocyte Biology, 74, 25–32. 12832439 10.1189/jlb.0902436

[aos15247-bib-0167] Vallejo, A.N. , Mueller, R.G. , Hamel, D.L.F. , Way, A. , Dvergsten, J.A. , Griffin, P. et al. (2011) Expansions of NK‐like T cells with chronologic aging: novel lymphocyte effectors that compensate for functional deficits of conventional NK cells and T cells. Ageing Research Reviews, 10, 354–361. 20932941 10.1016/j.arr.2010.09.006 PMC3039714

[aos15247-bib-0168] Van Acker, H.H. , Capsomidis, A. , Smits, E.L. & Van Tendeloo, V.F. (2017) CD56 in the immune system: more than a marker for cytotoxicity? Frontiers in Immunology, 8, 892. 28791027 10.3389/fimmu.2017.00892 PMC5522883

[aos15247-bib-0169] van Beek, J.H.G.M. , Kirkwood, T.B.L. & Bassingthwaighte, J.B. (2016) Understanding the physiology of the ageing individual: computational modelling of changes in metabolism and endurance. Interface Focus, 6, 20150079. 27051508 10.1098/rsfs.2015.0079 PMC4759747

[aos15247-bib-0170] Vingerling, J.R. , Dielemans, I. , Hofman, A. , Grobbee, D.E. , Hijmering, M. , Kramer, C.F.L. et al. (1995) The prevalence of age‐related maculopathy in the Rotterdam Study. Ophthalmology, 102, 205–210. 7862408 10.1016/s0161-6420(95)31034-2

[aos15247-bib-0171] Wagner, B.D. , Patnaik, J.L. , Palestine, A.G. , Frazer‐Abel, A.A. , Baldermann, R. , Holers, V.M. et al. (2021) Association of systemic inflammatory factors with progression to advanced age‐related macular degeneration. Ophthalmic Epidemiology, 29, 139–148. 33827374 10.1080/09286586.2021.1910314 PMC8497647

[aos15247-bib-0172] Walker KA , Ficek BN & Westbrook R (2019): Understanding the role of systemic inflammation in Alzheimer's disease. 10.1021/acschemneuro.9b00333 31241312

[aos15247-bib-0173] Weng, N.‐P. , Akbar, A.N. & Goronzy, J. (2009) CD28(‐) T cells: their role in the age‐associated decline of immune function. Trends in Immunology, 30, 306–312. 19540809 10.1016/j.it.2009.03.013 PMC2801888

[aos15247-bib-0174] Wong, T. , Chakravarthy, U. , Klein, R. , Mitchell, P. , Zlateva, G. , Buggage, R. et al. (2008) The natural history and prognosis of neovascular age‐related macular degeneration a systematic review of the literature and meta‐analysis. Ophthalmology, 115, 116–126. 17675159 10.1016/j.ophtha.2007.03.008

[aos15247-bib-0175] Wong, W.L. , Su, X. , Li, X. , Cheung, C.M.G. , Klein, R. , Cheng, C.‐Y. et al. (2014) Global prevalence of age‐related macular degeneration and disease burden projection for 2020 and 2040: a systematic review and meta‐analysis. Lancet Global Health, 2, e106–e116. 25104651 10.1016/S2214-109X(13)70145-1

[aos15247-bib-0176] Xu, X. , Ritz, B. , Coleman, A.L. , Liew, Z. , Deapen, D. , Lee, E. et al. (2021) Non‐steroidal anti‐inflammatory drug use and risk of age‐related macular degeneration in the California Teachers Study. Drugs & Aging, 38, 817. 34309807 10.1007/s40266-021-00885-z PMC8419134

[aos15247-bib-0177] Zhang, Z.Y. , Bao, X.L. , Cong, Y.Y. , Fan, B. , Li, G.Y. & Žerovnik, E. (2020) Autophagy in age‐related macular degeneration: a regulatory mechanism of oxidative stress. Oxidative Medicine and Cellular Longevity, 2020, 2896036. 32831993 10.1155/2020/2896036 PMC7429811

